# Stereochemical bias introduced during RNA synthesis modulates the activity of phosphorothioate siRNAs

**DOI:** 10.1038/ncomms7317

**Published:** 2015-03-06

**Authors:** Hartmut Jahns, Martina Roos, Jochen Imig, Fabienne Baumann, Yuluan Wang, Ryan Gilmour, Jonathan Hall

**Affiliations:** 1 Department of Chemistry and Applied Biosciences, ETH Zürich, Vladimir-Prelog-Weg-4, CH-8093 Zürich, Switzerland; 2 Institute for Organic Chemistry, Westfälische Wilhelms-Universität Münster, D-48149 Münster, Germany

## Abstract

An established means of improving the pharmacokinetics properties of oligoribonucleotides (ORNs) is to exchange their phosphodiester linkages for phosphorothioates (PSs). However, this strategy has not been pursued for small interfering RNAs (siRNAs), possibly because of sporadic reports that PS siRNAs show reduced inhibitory activity. The PS group is chiral at phosphorous (*R*p/*S*p centres), and conventional solid-phase synthesis of PS ORNs produces a population of diastereoisomers. Here we show that the choice of the activating agent for the synthesis of a PS ORN influences the *R*p/*S*p ratio of PS linkages throughout the strand. Furthermore, PS siRNAs composed of ORNs with a higher fraction of *R*p centres show greater resistance to nucleases in serum and are more effective inhibitors in cells than their *S*p counterparts. The finding that a stereochemically biased population of ORN diastereoisomers can be synthesized and exploited pharmacologically is important because uniform PS modification of siRNAs may provide a useful compromise of their pharmacokinetics and pharmacodynamics properties in RNAi therapeutics.

## Abstract (web summary)

Therapeutic oligonucleotides can be made more stable by substituting their achiral phosphodiester groups for chiral phosphorothioate linkages. Here, the authors present a synthesis of phosphorothioated RNAs, where the activator controls strand stereochemistry, and also the activity of assembled siRNAs.

## Graphical abstract

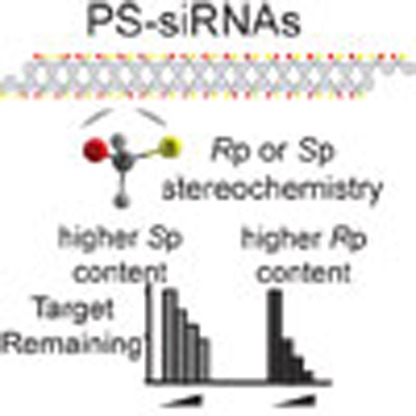

Chemically modified oligoribonucleotide (ORN) drugs represent an emerging class of pharmaceuticals of which, arguably, the most important is the small interfering RNAs (siRNA). siRNAs are duplexes composed of complementary ORNs of ~21 nucleotides (nt), with 2-nt overhangs. One of the ORNs (the ‘guide’ strand) directs the RNA-induced silencing complex (RISC) to cleave complementary mRNA targets and thereby represses gene expression^1^, whereas the other ORN (passenger strand) is discarded. For most applications *in vivo* the phosphodiester (PO) linkages of double-stranded siRNAs require protection against cleavage by exonucleases^2^,^3^,^4^. However, medicinal chemists have been unable to develop a readily-accessible uniform modification of siRNAs, which resists nuclease attack without sacrificing the reagent’s ability to trigger RISC-mediated degradation of its mRNA targets. Hence, still today, siRNAs are rarely used systemically in the absence of complex delivery formulations.

An effective means of protecting single-stranded RNA-based antisense drugs against nucleases is to exchange one of the non-bridging oxygens of each PO linkage with sulfur. Indeed, almost all single-stranded oligonucleotide drugs in clinical trials are phosphorothioates^5^. The phosphorothioate group (PS) is chiral, with either *R*p or *S*p configuration at phosphorous^6^. However, an efficient, practicable solid-phase chiral synthesis of PS ORNs using the H-phosphonate method or the phosphoramidite method is not yet available^7^,^8^ (reviewed in ref. 9) and PS ORNs are used as mixtures of 2^*n*^ diastereoisomers (*n*, number of PS linkages). PS diastereoisomers have distinct physical and biochemical properties and will interact differently with cellular RNAs and proteins (for example, see refs 10, 11, 12). Hence, the net pharmacological properties of PS ORN drugs likely represent the sum effect of the pharmacokinetic (PK) and pharmacodynamic (PD) properties of individual diastereoisomers in an isosequential population.

Full PS modification of siRNAs has been mostly neglected by the field after a few groups reported that PS siRNAs *in vitro* show reduced inhibitory activity compared with unmodified siRNAs^13^,^14^ and in some^15^,^16^ but not all^17^, cases toxicity. In this systematic study of the properties of uniformly modified PS siRNAs, we investigate whether the control of stereochemistry of PS ORN strands in a siRNA can be exploited to improve their pharmacological properties. We show here that the choice of the activating agent during synthesis of a PS RNA shifts the *R*p/*S*p ratio in the PS linkages throughout an ORN and when assembled into duplexes, this bias transforms the PK and PD activities of PS siRNAs. To our knowledge, this account represents the first demonstration of how a stereochemically biased population of PS siRNAs can be prepared and exploited to optimize its pharmacological profile. The findings of the study suggest that the PS modification of siRNA drugs may provide a useful compromise of PK and PD properties for *in vivo* applications. In addition, they encourage continued efforts towards the synthesis of full stereochemically pure PS ORNs.

## Results

### Tetrazole activators bias *R*p/*S*p ratios in PS dinucleotides

**Figure 1:**
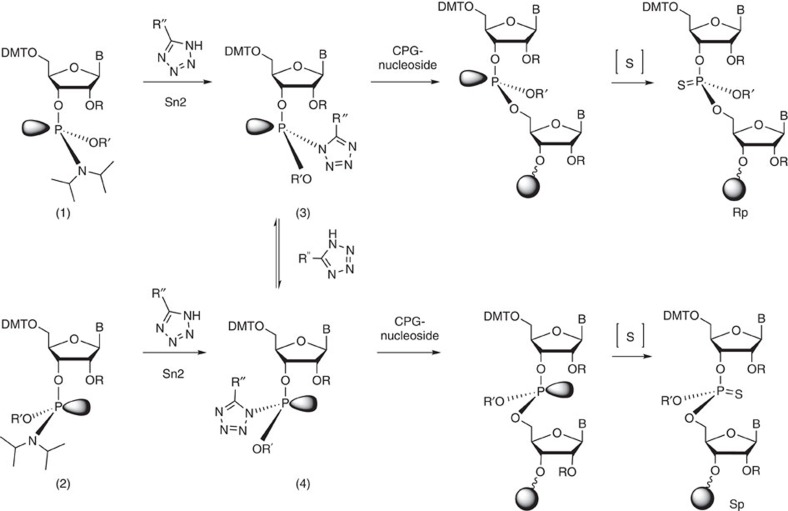
Mechanism and stereochemical course of phosphoramidite coupling during solid-phase ORN synthesis. Epimerization at P occurs at the tetrazolide stage (R, Si-*tert*-BuMe_2_; R', CH_2_CH_2_CN; R'', H or SCH_2_Ph; B, nucleobases adenine, guanine, cytosine and uracil; CPG, controlled pore glass solid support).

Solid-phase synthesis protocols of ORNs typically use tetrazole reagents to facilitate the attack of the ribose 5′-hydroxyl group on the activated phosphorous centre (Fig. 1)^18^. Tetrazoles act as weakly acidic and nucleophilic catalysts during the displacement of diisopropylamine at the P^III^-stereocentre by the 5′-hydoxyl group of the support-bound ribonucleoside. The final oxidative sulfurization step is stereoretentive, at least in the case of oligodeoxynucleotides (ODNs)^19^,^20^.

**Figure 2:**
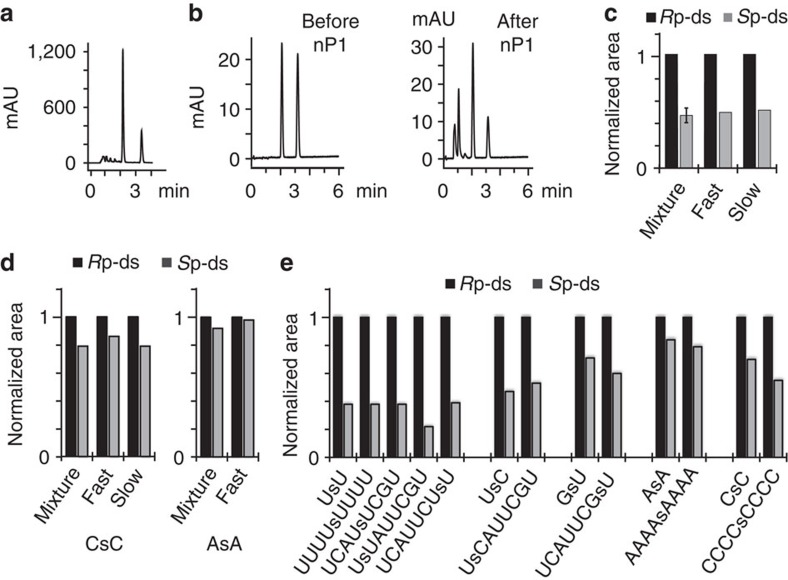
Properties of PS diribonucleotides. (**a**) HPLC chromatogram of BTT-synthesized UsU. (**b**) HPLC chromatogram of an equimolar mixture of UsU diastereoisomers before and after incubation with nP1. (**c**) Diastereoisomer ratios of BTT-synthesized UsU from epimeric (mixture) and from stereopure U-phosphoramidites: fast-migrating phosphoramidite isomer (fast) and slow-migrating isomer (slow). (**d**) Diastereoisomer ratios of BTT-synthesized CsC and AsA starting from epimeric (mixture) and stereopure phosphoramidites (fast-migrating phosphoramidite isomer (fast) and slow-migrating isomer (slow)). (**e**) Diastereoisomer ratios of BTT-synthesized octanucleotides containing single PS dinucleotide units. Error bars are s.d. of three independent experiments. Norm, normal.

We began the investigation with a study of PS diribonucleotides, following the example of a limited investigation using 2′-O-methoxyethyl (MOE) ribonucleotides^21^. We synthesized PS uridine dinucleotide (UsU) using standard 2′-O-tert-butyldimethylsilyl (TBDMS)-protected phosphoramidites and 5-benzylthio-1-H-tetrazole (BTT) as an activator. The diastereoisomeric products were separated using reverse-phase high-performance liquid chromatography (RP-HPLC), which showed an ~2:1 ratio of diastereoisomers (Fig. 2a). The major isomer was assigned the *R*p stereochemistry, as incubation of an equimolar mixture of isomers with nuclease P1 (nP1) attenuated selectively the slower migrating (*S*p) compound (Fig. 2b)^22^,^23^.

This product ratio was independent of the configuration of the starting materials as configurationally defined U-phosphoramidites **1** and **2** (Fig. 1; purified by column chromatography: Supplementary Figs 1–4, Supplementary Table 1) yielded the same 2:1 mixture (Fig. 2c), consistent with rapid epimerization at the P^(III)^ stereocentre via repetitive attack of the tetrazole before irreversible coupling of the ribonucleoside (Fig. 1)^24^,^25^. Analogous observations were made with CsC and AsA dinucleotides that were obtained in diastereoisomeric ratios of ~5:4 and 10:9, respectively (Fig. 2d). These stereochemical outcomes were mostly reproduced in longer sequences containing UsU, CsC and AsA units. Hence, the incorporation of UsU in the centre of U_8_, in the centre of a mixed-sequence octanucleotide, and at the 5′- or 3′-termini of octanucleotides yielded ~2:1 isomer ratios (Fig. 2e), as determined by RP-HPLC (Supplementary Tables 2–4).

**Figure 3:**
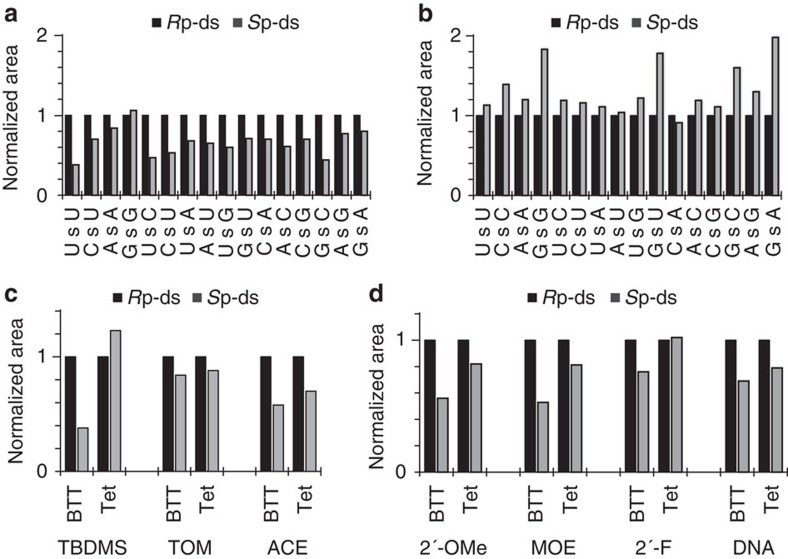
Diastereoisomer ratios of 2′-substituted PS dinucleotides synthesized with tetrazole activators. Ratios of PS diribonucleotides synthesized from TBDMS-protected phosphoramidites using BTT (**a**) and tetrazole (**b**). (**c**) and (**d**) Six UsU-containing PS dinucleotides synthesized from DNA (TsT) and from 2′-F-, 2′-*O*-methyl (OMe)-, MOE-, 2′-*O*-[(triisopropylsilyl)oxy]methyl (TOM)- and ACE-substituted phosphoramidites, using BTT and tetrazole (Tet). (Data from five dinucleotides shown in Fig. 2e is reshown in (**a**) and (**c**) for ease of comparison).

We then synthesized all possible 16 PS dinucleotides using two activators, BTT and tetrazole. For comparison, a third electron-deficient activator, dicyanoimidazole was investigated but gave unsatisfactory product yields. We determined the diastereoisomeric ratios of the products using the nP1/RP-HPLC method (Supplementary Fig. 5). For BTT the respective *R*p isomer was the dominant product in all cases except GsG (Fig. 3a, Supplementary Table 2), whereas for tetrazole the trend was reversed, that is, the *S*p isomers were the major products (Fig. 3b, Supplementary Table 3). The difference in the product profiles may represent different states of the thermodynamic equilibria between tetrazolides **3** and **4** before coupling; however, a kinetic resolution of the rapidly equilibrating **3** and **4** following a Curtin–Hammett principle cannot be discounted (Fig. 1). To further investigate the importance of the 2′-substituent on the *R*p/*S*p product ratio, we synthesized six UsU-containing PS dinucleotide from DNA and from 2′-F-, 2′-*O*-Me-, MOE-, 2′-*O*-[(triisopropylsilyl)oxy]methyl (TOM)- and 2′-*O*-bis(2-acetoxyethoxy)methyl (ACE)-substituted phosphoramidites, using BTT and tetrazole. In each case we assumed that the stereochemical outcome of the coupling reaction was independent of the *R*p/*S*p isomeric mixture of the starting phosphoramidites, as shown for 2′-*O*-TBDMS ribonucleotides (Fig. 2c,d) and for tetrazole-mediated coupling of DNA^26^.

The 2′-*O*-TOM protecting group, which presents a relatively low steric hindrance to phosphoramidite coupling^27^, produced a marginal and similar selectivity for the *R*p isomer from both the activators (Fig. 3c), suggesting that indeed steric crowding may contribute to reaction diastereoselectivity for the 2′-*O*-TBDMS derivatives. Results with the 2′-F and DNA phosphoramidites appeared to support this hypothesis (Fig. 3d). The electronic and conformational properties of the 2′-F-deoxyribose resemble those of RNA. However, the small fluoride minimizes steric hindrance for phosphoramidite coupling. Here BTT-mediated reaction produced a slight excess of the faster HPLC-running isomer (presumably *R*p), whereas tetrazole was unselective. In the case of DNA, which differs from RNA by its C2′-endo conformation and its lack of a 2′-substitutent on ribose, BTT and tetrazole produced similarly weak *R*p-selectivity (Fig. 3d). For the three 2′-*O*-alkyl substituents (2′-*O*-Me, 2′-MOE, 2′-*O*-ACE) stereoselectivites midway between those of 2′-*O*-TBDMS and 2′-*O*-TOM were observed. We also synthesized all 16 dinucleotides of DNA and 2′-*O*-Me using BTT, as well as four dinucleotides with the both activators to confirm that the product ratios for TsT and 2′-*O*-Me UsU were not outliers for the dinucleotide series and were highly reproducible (Supplementary Fig. 6, Supplementary Note 1).

For the coupling of a single nucleotide in a PS ORN the difference in the stereochemical ratios arising from the use of the two activators is small. For example, AsA synthesized using BTT has an *R*p/*S*p ratio of ~1.2, whereas with tetrazole this ratio is reduced to ~0.8 (Fig. 3a,b); for tetrazole especially, diastereoselective excesses only exceeded 20% for 6 of the 16 dinucleotides (Fig. 3b). Thus, efforts to model such small differences of this solid-phase reaction would be very difficult. However, in an ORN containing 20 PS linkages these small differences are at least partially cumulative and therefore might affect considerably the bulk biophysical properties of an isosequential population of diastereoisomers. To our knowledge, there are no reports on how the physicochemical and biological properties of PS ORNs may be influenced by a variation in the relative population of individual diastereoisomers.

### Higher *R*p content of PS siRNAs improves cellular activity

To assess the biophysical and biological consequences of the use of different activators during the synthesis of PS ORNs, we prepared 24 PS ORNs and assembled siRNA sequences of various compositions targeting *TP53* (p53), *LIN28B* (Lin28), *TGFB1* (TGFβ1), *TGFB2* (TGFβ2), *RBFOX2* (Fox2) and *Renilla* luciferase (Ren; Table 1, Supplementary Table 5). SiRen was prepared ‘blunt-ended’.

Assuming that the stereochemical outcome in each coupling step depends only on the sequence of the newly-formed terminal dinucleotide unit (Fig. 2e) and reflects the diasteromeric ratio observed for isolated dinucleotide formation, a theoretical total *R*p content can be calculated for each PS siRNA by dividing the sum of the individual *R*p ratios for all the dinucleotide units (Fig. 3a,b) by the number of nucleotide linkages (Table 1). We then measured the melting temperatures (T_m_) of the duplexes (Supplementary Figs 7–10). It has been reported that short ODNs^11^ and ORNs composed of stereopure *R*p strands form more stable duplexes with complementary PO ORNs than their *S*p analogues, or even their PO counterparts^7^,^28^. In our case, the PO siRNAs showed the highest T_m_s of the three formats (Table 1). The T_m_s of all siRNAs synthesized using BTT (PS_BTT_ siRNA) were 5–6 °C higher than those of siRNAs synthesized using tetrazole (PS_tet_ siRNA). These data are consistent with a higher *R*p content (~60%) of the PS_BTT_ siRNA and support our hypothesis that the stereoselectivity observed during the formation of isolated dinucleotides (Fig. 3a,b) extends to the synthesis of ~21-nt ORNs.

**Figure 4:**
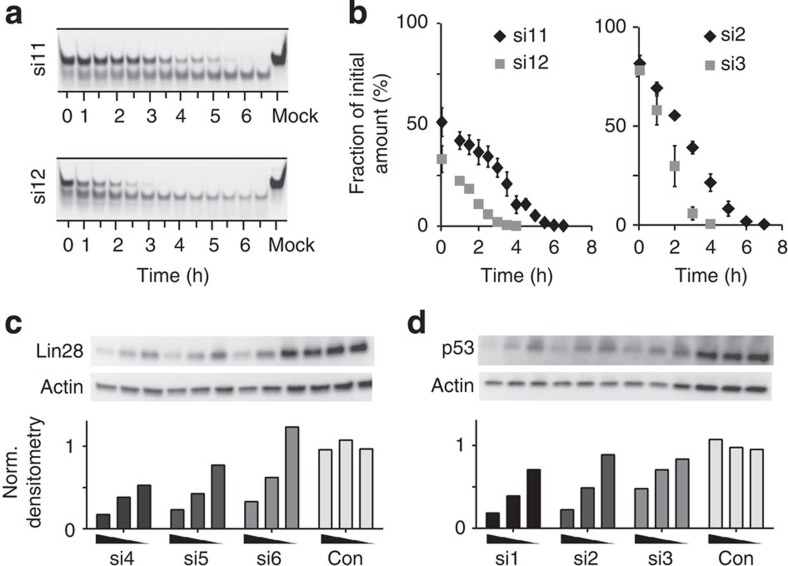
Pharmacological properties of PO- and PS- siRNAs. (**a**) Stability of PS_BTT_-synthesized (si11) and PS_tet_-synthesized (si12) siTGFβ2 siRNAs to nuclease degradation in FBS: ‘mock’ refers to no FBS treatment. (**b**) Quantification of blots in **a** and Supplementary Fig. 14 using the mock lane for normalization. Sequence-specific inhibition by PO and PS siRNAs of Lin28 protein in Huh7 cells (**c**) (24, 6, 2.5 nM), and p53 protein in HeLa cells (**d**) (6, 2.5, 0.6 nM). Error bars are s.d. of three independent experiments. Con, control; Norm, normalized.

The two most important pharmacological properties of an siRNA are its stability to nucleases and its potency. We therefore tested the effects of stereochemical bias in PS siRNAs on their stability to nucleases in serum and on their biological activity in cells. Stability of ORNs to serum nucleases is commonly assayed using mammalian sera such as fetal bovine serum (FBS). We incubated BTT-synthesized PS and PO siRNAs targeting Fox2, p53, transforming growth factor β1 (TGFβ1) and TGFβ2 with a solution of 10% FBS in a time-course experiment under conditions to highlight the differences in stabilities to nucleases. We quenched the reactions at regular intervals and analysed the equally loaded mixtures on a non-denaturing gel. Under our conditions the PS siRNAs showed superior stability to their PO counterparts in all the cases (Supplementary Figs 11–14). In two cases (TGFβ2 and p53), we compared the stability of the PS_BTT_ and PS_tet_ siRNAs under identical incubation conditions in a detailed time-course experiment (Fig. 4a,b).

Stability of single-stranded PS ODNs to nucleases in sera is reportedly greater for *S*p- than for *R*p isomers^29^,^30^. Also, double-stranded ODNs and ORNs are more resistant than their single-stranded counterparts^17^,^31^. We assigned the rapid appearance of faster migrating (lower) bands for all of the siRNAs to their nuclease-mediated degradation. The PS_BTT_ siRNAs si2 and si11 demonstrated higher stability than si3 and si12, respectively, over the long term. This was perhaps due to the higher thermal stability of the *R*p-biased duplexes, in agreement with previous suggestions that duplexes of higher stability demonstrate greater nuclease resistance^31^.

To compare the potency of the three classes of siRNAs, we tested them at graded concentrations for their inhibition of target proteins Lin28 and p53 in Huh7 and HeLa cells, respectively (Fig. 4c,d; Supplementary Fig. 15). The negative control (Con), a sequence-unrelated siRNA^32^, was inactive. PO siRNAs (si1 and si4) inhibited their targets by 80% at the highest treatment concentrations. Surprisingly, both the PS_BTT_ siRNAs (si2 and si5) appeared to inhibit their targets more efficiently than the PS_tet_ siRNAs (si3 and si6), albeit to a lower extent than their PO counterparts (some toxicity was observed at the highest concentrations). In other words, the higher content of *R*p linkages in the PS_BTT_ siRNA was associated with increased potency.

**Figure 5:**
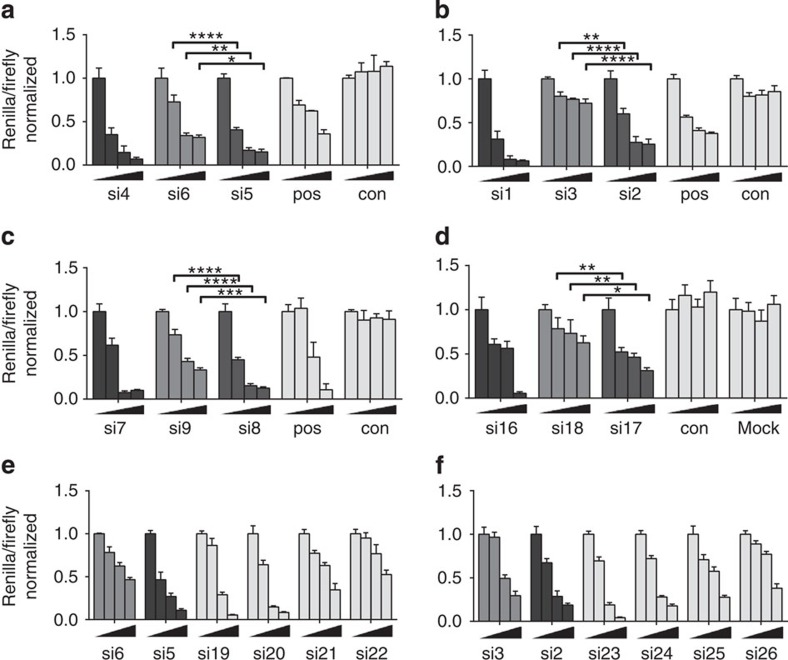
Sequence-specific target inhibition of *Renilla*/firefly luciferase reporters in HeLa cells. Negative and positive controls (con and pos, respectively), PO- and PS- siRNAs (Table 1) targeting (**a**) Lin28, (**b**) p53, (**c**) TGFβ1 and (**d**) Ren were co-transfected with luciferase reporter vectors into HeLa cells at increasing concentrations (0.4, 1.5, 6, 24 nM for (**a**,**b**,**d**); 0.3, 1, 4, 16 nM for **c**). *Renilla* luminescence values were normalized against the reference firefly luminescence and then to the value from the lowest treatment concentration. Error bars are s.d. of three transfections. Mixed backbone PO/PS siRNAs (Table 2) were tested against Lin28 (**e**) and p53 (**f**) under identical conditions. (*)*P*<0.05; (**)*P*<0.01; (***)*P*<0.001; (****)*P*<0.0001.

Quantification of bands on western blots cannot be relied upon to determine small differences in the activity. A routine and sensitive method to compare the activity of siRNAs is the luciferase reporter assay, in which a target site for an siRNA can be cloned^32^,^33^. We employed six luciferase reporter genes, of which five contained a cloned target site for siRNAs of Table 1 (Supplementary Note 2), and one in which an unmodified *Renilla* reporter plasmid was used. SiRNAs were tested at four concentrations. In 5/6 cases the PO siRNAs (si1, si4, si7, si16 and si27) were the most effective, as expected (Fig. 5a–d; Supplementary Fig. 16). Strikingly, however, the PS_BTT_ siRNAs (higher *R*p content; si2, si5, si8, si17, si15 and si29) showed roughly twofold higher activity than their PS_tet_ siRNA analogues (higher *S*p content).

Many factors affect the potency of siRNAs and include strand selection^34^,^35^, duplex stability^34^,^36^, the presence of nucleotide motifs^37^ and chemical modification of guide and/or passenger strands^38^. For unmodified siRNAs, it is known that specific PO cleavage in the centre of the passenger strand by the RNase H-like Argonaute2 (Ago2) is an important step in the activation of the guide strand^10^,^39^,^40^. This was elegantly demonstrated employing a PS linkage at the said position: the use of an *R*p PS linkage partially attenuated, whereas an *S*p PS group greatly attenuated passenger strand cleavage^10^. Our own data are consistent with these observations as the higher *R*p PS content in the central position of passenger strands of PS_BTT_ siRNAs correlates with higher potency (Fig. 5a–d).

To shed additional light on the roles of strand modifications, duplex stability and PS stereochemistry in the potency of PS siRNAs, we assembled Lin28 and p53 siRNAs, combining a PO ORN with either a PS_BTT_ or a PS_tet_ ORN (Table 2). We measured their T_m_s and tested them in cells in pair-wise comparisons.

For all pairs of siRNAs (si19/si21, si20/si22, si23/si25 and si24/si26), switching the PS backbone from the guide to the passenger strands did not significantly change the T_m_. However, in each case the potency was clearly highest for siRNAs with a PO guide strand (si19, si20, si23 and si24), regardless of the stereochemistry of the PS passenger strands (Fig. 5e,f). The results suggest that an unmodified guide strand is the dominating factor in the activity of a mixed PO/PS siRNA, consistent with numerous previous accounts on modifications to siRNAs^31^,^41^. Finally, we compared the properties of two pairs of siRNAs with a PS guide strand and a PO passenger strand (si21/22 and si25/26). The chimeric siRNAs bearing PS_BTT_ guide strands (si21 and si25) showed the higher T_m_s (3.0–3.3 °C), as well as a superior potency to those comprising PS_tet_ strands (si22 and si26). This is consistent with the properties of their fully phosphorothioated analogues (Table 1, Fig. 5a–d). Furthermore, it demonstrates that a bias for PS linkages with *R*p stereochemistry also in the PS guide strand is beneficial for potency, in analogy to the effects described for the RNase H-mediated cleavage of mRNAs by stereopure *R*p antisense PS ODNs^11^. Recently, two crystal structures of human Ago2 in complex with RNA guides were published^42^,^43^. Both the structures show extensive hydrogen bonding contacts between Ago2 residues and the non-bridging oxygens of all PO linkages at the 5′-end and with one PO group at the 3′-end of the guide^42^,^43^. Given the multitude of these interactions it is to be expected that thiolation of POs in these regions of the guide will affect its interactions with Ago2 and therefore also the properties of PS siRNAs in RISC.

### A high *R*p content of PS siRNAs increases loading into RISC

**Figure 6:**
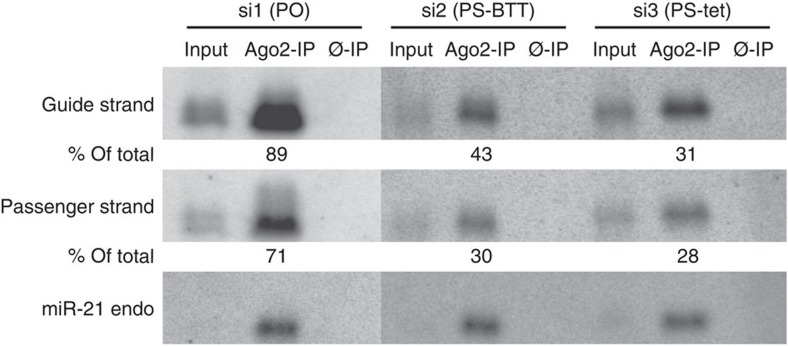
Composition of the RISC/Ago2 in HeLa cells transfected with PS- siRNAs. p53 PO-, PS_BTT_- and PS_tet_-siRNAs (si1, si2, si3, respectively; 30 nM) were transfected into HeLa cells and worked up under identical conditions after 24 h. Northern blot detection using ^32^P-labelled respective counter strands: guide and passenger strands of si1, si2 and si3 (see Supplementary Fig. 17 for original blot). RNA samples loaded were each input RNA (5% of total), Ago2 and control (Ø-IP) immunoprecipitations (47.5% each of total). Endogenously-expressed miR-21 detection served as a loading control. Densitometric quantification of Ago2-IP (in % of total input) is shown. One representative blot of the two independent experiments is shown (see Supplementary Fig. 17).

To add further insight on the interactions of PS siRNAs with RISC, we transfected HeLa cells with PO, PS_BTT_ and PS_tet_ siRNAs (si1, si2 and si3, respectively) and immunoprecipitated the RISC using an Ago2 antibody. We then performed northern blotting for the presence of the individual guide and passenger strands in the complex, as described previously^44^. For the wild-type siRNA (si1), the great majority of the siRNA strands were present in complex with Ago2 compared with the input sample (Fig. 6, Supplementary Fig. 17). In line with the aforementioned study, we assumed that the northern blotting detected individual passenger and guide strands (and not siRNA) of si1 because Ago2 rapidly cleaves the passenger strands of siRNAs^44^, and also because the affinity of Ago2 for single-stranded RNA is much higher than for double-stranded siRNA^45^. Consistent with the high potency of si1, the guide strand appeared to occupy a much larger fraction in RISC than the passenger, although the affinity of the two northern probes for the guide and the passenger strands may not be equal.

As expected, bands on the blot were much weaker for the PS_BTT_ and PS_tet_ ORNs, possibly because of an overall lower binding affinity of the radiolabelled probes for PS ORNs. For both the PS siRNAs, loading of the siRNA into RISC was incomplete, as seen by residual bands in the input samples. However, a larger proportion (43%; Fig. 6) of the PS_BTT_ guide strand of si2 was taken into the Ago2 complex from the input sample compared with the PS_tet_ guide strand of si3 (31%; Fig. 6). This is consistent with the higher potency of si2 over si3 against both endogenous p53 protein (Fig. 4d) and the luciferase reporter genes (Fig. 5).

Taken together, our results suggest that an *R*p bias in both the PS guide and passenger strands contributes to the increased potency of uniformly modified PS_BTT_ siRNAs. The origin of the effects is possibly advantageous interactions with Ago2, both during passenger strand cleavage/dissociation, uptake of the guides into RISC and guide-mediated mRNA cleavage. The greater stability to nucleases shown by the PS_BTT_ siRNAs may also play a contributory role.

## Discussion

Introduction of the PS modification was an enabling advance in the field of oligonucleotide therapeutics. However, the chirality at the new internucleotide linkage was an unwelcome complication and despite considerable research, a practicable solid-phase synthesis of stereopure PS oligonucleotides is still unavailable. The pharmacological consequences of uniformly modifying siRNAs with PS groups has not been investigated in detail, probably because of sporadic reports that PS siRNAs *in vitro* are less active than unmodified siRNAs^13^,^14^. However, medicinal chemists are well aware that small reductions in the potency of a drug can be easily compensated by an improved PK profile.

Here we have confirmed that distinct tetrazole activators produce a stereogenic bias during the solid-phase synthesis of PS ORNs. In a siRNA composed of two ORNs and ~40 PS linkages, this bias substantially influences the biophysical and biological properties of the duplex. Specifically, compared with tetrazole, ribonucleoside coupling catalysed by BTT yields a higher fraction of *R*p PS linkages in an ORN, as evidenced by elevated T_m_s. Surprisingly, this produces a more potent PS siRNA, albeit with slightly less activity than its PO counterpart. It is likely that other commonly used activating agents, for example, ethylthiotetrazole, may similarly alter these properties and thus, they also deserve to be investigated.

Controlling the configuration of phosphorous centres has been of central importance in various studies charting the stereochemical course of enzymatic processes. Similarly, we envisage that understanding the synergy and additivity of this motif in a supra(bio-)molecular context may be valuable in the field of siRNA therapeutics. The use of PS_BTT_ siRNAs in animal models of disease is currently under investigation.

## Methods

### General

Solvents for purification and chromatography were purchased as technical grade. For column chromatography SiO2-60 (230–400-mesh ASTM; Fluka) was used as the stationary phase. Analytical thin layer chromatography was performed on aluminium plates precoated with SiO_2_-60 F254 (Merck) and visualized with a ultraviolet lamp (254 nm). Concentration *in vacuo* was performed at ~10 mbar and 40 °C, drying at ~10^−2^ mbar and room temperature (rt). NMR (nuclear magnetic resonance) spectra were measured on a Bruker AV400 spectrometer at rt. ^31^P NMR spectra are reported as follows: chemical shift in parts per million in CDCl_3_ and CD_3_CN.

### Synthesis of ORNs

Chemicals for oligonucleotide synthesis were purchased from Aldrich and TCI (Sigma-Aldrich Chemie GmbH, D-89555 Steinheim). Phosphoramidites were obtained from Thermo Fisher Scientific (Waltham, MA), Glen Research (TOM-U) (Sterling, VA) and from Dharmacon (ACE-U). The activator 5-benzylthiotetrazole (BTT) was purchased from Biosolve (5555 CE Valkenswaard, the Netherlands). All oligonucleotides used in this work were synthesized with a MM12 synthesizer from Bio Automation Inc. (Plano, TX) on 500 Å UnyLinker CPG from ChemGenes (Wilmington, MA). The coupling time for phosphoramidites was 2 × 90 s. The oligonucleotides were purified on an Agilent 1200 series preparative HPLC fitted with a WatersXBridge OST C-18 column, 10 × 50 mm, 2.5 μm at 60 °C. The RNA phosphoramidites were prepared as 0.08 M solutions in dry acetonitrile (ACN), the activator BTT (Biosolve BV, 5555 CE Valkenswaard, the Netherlands) was prepared as a 0.24 M solution dry ACN, the activator 1H-tetrazole (Tet; Sigma-Aldrich Chemie GmbH, D-89555 Steinheim) was purchased as a 0.45 M solution in dry ACN. Oxidizer was prepared as a 0.02 M I_2_ solution in THF/Pyridine/H_2_O (70:20:10, w/v/v/v); the sulfurizing reagent was prepared as a 0.05 M solution of 3-((*N*,*N*-dimethylaminomethylidene)amino)-3H-1,2,4-dithiazole-5-thione (DDTT; Sulfurizing Reagent II; Glen Research, Virginia) in dry pyridine/ACN (60:40). Capping reagent A was: THF/lutidine/acetic anhydride (8:1:1) and capping reagent B was: 16% *N*-methylimidazole/THF. Deblock solution was prepared as a 3% dichloro acetic acid in dichloroethane. The cleavage, the deprotection of the bases and phosphordiesterbackbone was done by the incubation of the CPG-support for 2 h, at 65 °C, 1.8 bar in gaseous methylamine. Deprotection of 2′-O-TBDMS (tert-butyldimethylsilyl-) was performed by incubation of the ORN for 1.5 h, at 70 °C in a mixture of dry 1-*N*-methyl-2-pyrrolidone/triethylamine/triethylamine x 3HF.

RP-HPLC purification of ORNs. Running buffer for HPLC purification of single-stranded ORNs (up to 23 nt): buffer A (0.1 M triethylammonium acetate), buffer B (methanol); gradient for the DMT-on purification: 20–60% buffer B over 5 min; gradient for the DMT-off purification: 5 –35% buffer B over 5 min. Fractions containing the product were collected and dried in a miVac duo SpeedVac from Genevac. The oligonucleotides were analysed by LC-MS (Agilent 1200/6130 system) on a Waters Acquity OST C-18 column, 2.1 × 50 mm, 1.7 μM, 65 °C. Buffer A: 0.4 M HFIP, 15 mM triethylamine; buffer B: MeOH. Gradient: 7–35% B in 14 min; flow rate: 0.3 ml min^−1^.

### Nuclease degradation assays

To determine the stereochemistry of PS diastereoisomers, an equal quantity of both the diastereoisomers were mixed and a sample was injected onto HPLC to generate a reference chromatagram. Then mixtures of ZnCl_2_ (2.5 μl, 5 mM), NaOAc (10 μl, 20 mM), H_2_O (6.5 μl; Milli-Q) and 5 μl of the RNA-dinucleotide diastereoisomers (1 mM) were prepared. nP1 1 μl (2.4 U μl^−1^) was added and the samples were incubated at 37 and 50 °C (UsC, UsA, CsU, CsA and GsA) for 30 min. The reaction was stopped by adding EtOH/HCl (9:1, 5 μl). To denaturate the nP1 enzyme the samples were heated to 95 °C for 3 min. To separate the enzyme from the ORNs the samples were extracted with CHCl_3_ (20 μl). Supernatant (20 μl) was injected and analysed by HPLC.

### Stability of siRNA in 10% FBS

Solution of the siRNA (25 μl, 50 μM) was diluted by addition of 42.5 μl H_2_O (Milli-Q) and mixed with 7.5 μl FBS, Sigma-Aldrich, Buchs, CH). The reaction mixture was incubated at 37 °C (controls were treated identically, except FBS solution was replaced by water). At specific time points aliquots of 6 μl were collected, flash frozen in liquid nitrogen and stored at −80 °C. Samples were analysed on a 15% non-denaturing acrylamide gel. Gels were prepared using 30% acrylamide solution diluted with water (Millipore) and 5 × Tris–Borate–EDTA buffer, 10% ammonium persulfate solution to start polymerization. DNA loading buffer II was added to the samples without heating before loading onto the gel. The gel was run for 20 min at 50 V followed by 1 h at 90 V. After electrophoresis the gel was stained with GelRed nucleic acid stain (incubation time: 10 min) and analysed on a Bio-Rad Gel DocTMOR System. All samples were assayed in triplicates (Supplementary Figs 11–14).

### Cell culture and transfection of siRNAs

HeLa cells (ATCC, no. CCL-2; LGC, Molsheim, FR) and Huh7 cells (Cell Lines Service, no. 300156, Eppelheim, DE) were maintained in Dulbecco's Modified Eagle's medium (Gibco, Invitrogen, Basel, CH) supplemented with 10% FBS (Sigma-Aldrich, Buchs, CH). SiRNA against *Renilla* (siRen: Supplementary Table 5) was from Dharmacon (Chicago, USA) and the control siRNA (Con; no. AM4640) was from Ambion (Austin, USA). RNAs were transfected using Oligofectamine (no. 12252-011, Invitrogen, Basel, CH) according to the manufacturer's instructions.

### Reporter plasmid preparation and sequences

PsiCHECK2 (no. C8021, Promega, Dübendorf, CH) dual luciferase reporter plasmids were cut with Not1 and Xho1 restriction enzymes, and the inserts were cloned into the 3′-untranslated region of the Renilla gene to yield reporter constructs having fully complementary binding sites for the siRNA. The inserted sequences in the psiCHECK2 vector are reported in Supplementary Note 2. The psiCHECK2 vectors were sequenced and subsequently transfected as described above. For reporting the siRen activity, an empty PSICHECK2 luciferase plasmid was used.

### Luciferase assays

HeLa cells were seeded in white 96-well plates and the RNAs were transfected after 4 h. There were no overt differences in cell viability between the cells transfected with PO siRNA and the control cells. All transfections were performed in triplicates. After 24 h DNA plasmid (20 ng per well) was transfected using jetPEI (no. 101-10, Polyplus, Illkirch, FR) according to the manufacturer's protocol. After 48 h, the supernatants were removed and the firefly substrate (15 μl; Dual-GloR Luciferase Assay System, Promega, Dubendorf, CH) that was diluted with 15 μl H_2_O was added. Luminescence was measured on a microtitre plate reader (Mithras LB940, Berthold Technologies, Bad Wildbad, DE). After measurement 15 μl of *Renilla* substrate per well was added and the measurement was repeated. Values were normalized against firefly luciferase and the lowest siRNA concentration transfected. All statistical analyses were performed by ANOVA using Dunnett's post-test. All statistics were run with GraphPad.

### Western blotting

Cells were lysed with radioimmunoprecipitation assay lysis buffer (R 0278; Sigma) for p53 detection and with lysis buffer (10 mM Hepes, 400 mM NaCl, 3 mM MgCl_2_, 0.5% Triton-X 100, 1 mM DTT and 10% Glycerol) for LIN28B detection. Protein concentrations were determined using a BCA assay (Thermo Fisher Scientific 23225), the protein (10–20 ng) was mixed with equal quantities of SDS loading buffer (100 mM Tris–HCl, 4% SDS, 20% glycerol and 0.2% bromophenol blue). Samples were heated at 95 °C for 5 min, separated on SDS gels and transferred to polyvinylidene difluoride membranes. Non-specific membrane binding was blocked for 40 min at rt with 5% or milk in phosphate-buffered saline containing 0.05% Tween-20. Membranes were incubated overnight at 4 °C with appropriate primary antibodies p53 (no. sc-126) from Santa Cruz Biotechnology, Lin28B (no. A5316) from Cell Signaling Technology and β-ACTIN (no. A5316) from Sigma Life Science. After washing, the membranes were incubated with horseradish peroxidase-conjugated secondary antibodies for 2 h at rt in blocking buffer and washed again. Signals generated by the chemiluminescent substrate (ECL(+); Amersham Biosciences) were captured by a cooled CCD (charge-coupled device) camera (Bio-Rad). Protein bands were quantified by densitometry using the analysis software imageJ. Samples were normalized against β-ACTIN protein band and the average of transfection with control siRNA Con.

### RISC affinity assays

SiRNAs si1, si2 and si3 were reverse-transfected with RNAiMax (Life Technologies) according to the manufacturer’s recommendation at 30 nM final concentration into HeLa cells in 15 cm cell culture dishes (~1.2 × 10^7^ cells; 60% confluency) and incubated for 24 h. Ago2 immunoprecipitation was performed with modifications as described previously^46^. In brief, PBS-washed cells were collected and lysed in NP40 lysis buffer (50 mM HEPES pH 7.5, 150 mM KCl, 0.5% NP40, 0.5 mM DTT, 2 mM EDTA and 50 U ml^−1^ RNAsin, complete protease inhibitor (Roche)). Each cleared lysate was equally divided for Ago2 (clone 9A11, Ascenion, Munich, Germany) and control IP samples (non-specific rat serum IgG, (Sigma)). Five per cent (of 1st and 2nd replicate) of each input sample was collected for later RNA extraction. Then, 40 μl of Prot G Dynabeads (Life Technologies) per ½ 15 cm dish were washed two times with 1 ml of citrate-phosphate buffer (25 mM citric acid, 66 mM Na2HPO4 and pH 5.0). The Ago2 or rat IgG control serum antibodies (20 μg per 40 μl of Prot G Dynabeads) were immobilized in a total volume of 1,000 μl of citrate-phosphate buffer by gentle rolling for 1 h at 4 °C. Thereafter, the beads were washed three times with each 1 ml of NP40 lysis buffer and blocked for 1 h at 4 °C with 1 ml of NP40 lysis buffer containing BSA (10 μg ml^−1^). The beads were washed three times with 1 ml of NP40 lysis buffer and resuspended in 50 μl therein. Lysates were incubated with the Ago2/control antibody-coupled Prot G beads for 1 h at 4 °C with gentle rolling and then washed five times with 1 ml of IP wash buffer (50 mM HEPES, pH 7.5, 300 mM KCl, 0.05% NP40, 0.5 mM DTT and complete protease inhibitor (Roche)). Finally, bound RNA was released by the addition of 200 μl proteinase K digest buffer (100 mM Tris–HCl pH 7.5, 150 mM NaCl and 12.5 mM EDTA) containing 240–440 μg proteinase K (recombinant PCR grade solution, (Roche)) at 65 °C for 15 min shaking. RNA from Ago2/control IP and input was isolated by chloroform/phenol (Life Technologies) extraction and resuspended in 20 μl PCR grade water and subjected to northern blot analysis as described in ref. 47. One microgram of total input RNA and 10 μl (50%) of IP RNA were denatured in RNA loading buffer for 5 min at 95 °C and separated on a 15% denaturing polyacrylamide/urea gel electrophoresis. RNA transfer onto neutral nylon membrane (Hybond NX, GE Healthcare) and EDC/1-methylimidazole crosslinking was performed as described previously^47^. Detection probes were generated according to ref. 48. PO backbone ssRNA sip53 (2.5 μl each; 50 μM stock; guide or passenger strand) and 2′-*O*-Me anti-miR-21 as normalization control were 5′ phosphorylated using T4 phosphokinase, 3′-phosphatase-free (Roche) according to the providers’ protocol with 1 μl γ-^32^P-ATP (6000Ci/mmol Perkin-Elmer) followed by chloroform/phenol purification and used for the detection of the respective counter strand. Membranes were (pre-)-hybridized rotating at 40 °C overnight. Washed membranes were exposed to Phospho-Imager screen (GE Healthcare) for at least 24 h and the signals were detected by Typhoon scanner device FLA-7000 (GE Healthcare). Densitometric signal quantification was done by using ImageQuantTL software (GE Healthcare). Assays were performed in two independent biological replicates.

## Author contributions

J.H. and R.G. conceived the project; H.J., M.R., F.B., J.I. and Y.W. performed the experiments; H.J., R.G. and J.H. wrote the paper.

## Additional information

**How to cite this article:** Jahns, H. *et al*. Stereochemical bias introduced during RNA synthesis modulates the activity of phosphorothioate siRNAs. *Nat. Commun*, 6:6317 doi: 10.1038/ncomms7317 (2015).

## Supplementary Material

Supplementary Information Supplementary Figures 1-17, Supplementary Tables 1-5 and Supplementary Notes 1-2

## Figures and Tables

**Table 1:** Properties of PO- and PS siRNAs.

Target	Sequence of siRNA*	siRNA	Nt link†, (% *R*_p_‡)	*T*_m_ (^o^C)
p53	5′UUGUUUUCAGGAAGUAGUUUUUUAACAAAAGUCCUUCAUCAA	si1	PO	66.3
		si2	PS_BTT_ (62)	58.0
		si3	PS_tet_ (45)	52.7
Lin28	5′AAAUCCUUCCAUGAAUAGUTTTTUUUAGGAAGGUACUUAUCA	si4	PO	67.2
		si5	PS_BTT_ (56)	55.5
		si6	PS_tet_ (40)	50.6
TGFβ1	5′CCAACUAUUGCUUCAGCUCUUUUGGUUGAUAACGAAGUCGAG	si7	PO	74.1
		si8	PS_BTT_ (62)	67.0
		si9	PS_tet_ (44)	61.7
TGFβ2	5′GGAUUGAGCUAUAUCAGAUUUUUUCCUAACUCGAUAUAGUCUAA	si10	PO	70.8
		si11	PS_BTT_ (65)	63.0
		si12	PS_tet_ (47)	57.0
Fox2	5′CCUGGCUAUUGCAAUAUUUUUUUGGACCGAUAACGUUAUAAA	si13	PO	68.5
		si15	PS_BTT_ (62)	61.7
		si14	PS_tet_ (47)	55.0
Ren	5′GAGCGAAGAGGGCGAGAAAUUCUCGCUUCUCCCGCUCUUUAA	si16	PO	nd
		si17	PS_BTT_ (61)	nd
		si18	PS_tet_ (43)	nd

BTT, 5-benzylthio-1-H-tetrazole; Nt, nucleotide; PO, phosphodiester; PS, phosphorothioate; siRNA, small interfering RNA; tet, 1H-tetrazole; *T*_m_, melting temperature.

^*^Upper sequence: siRNA guide strand; lower sequence: passenger strand.

^†^Nucleotide linkages in both strands of the siRNAs: PS_BTT_, PS_tet_: PS ORNs synthesized with BTT and tetrazole, respectively.

^‡^Calculated *R*p content of siRNA.

**Table 2:** Melting temperatures of mixed backbone PS siRNAs.

siRNA	Sequence of siRNA*	Nt link† (% *R*_p_‡)	*T*_m_ (^o^C)
si19	5′AAAUCCUUCCAUGAAUAGUTTTTUUUAGGAAGGUACUUAUCA	PO	61.4
		PS_BTT_ (58)	
si20	5′AAAUCCUUCCAUGAAUAGUTTTTUUUAGGAAGGUACUUAUCA	PO	56.9
		PS_tet_ (40)	
si21	5′AAAUCCUUCCAUGAAUAGUTTTTUUUAGGAAGGUACUUAUCA	PS_BTT_ (50)	60.9
		PO	
si22	5′AAAUCCUUCCAUGAAUAGUTTTTUUUAGGAAGGUACUUAUCA	PS_tet_ (41)	56.9
		PO	
si23	5′UUGUUUUCAGGAAGUAGUUUUUUAACAAAAGUCCUUCAUCAA	PO	62.8
		PS_BTT_ (61)	
si24	5′UUGUUUUCAGGAAGUAGUUUUUUAACAAAAGUCCUUCAUCAA	PO	60.3
		PS_tet_ (46)	
si25	5′UUGUUUUCAGGAAGUAGUUUUUUAACAAAAGUCCUUCAUCAA	PS_BTT_ (63)	62.3
		PO	
si26	5′UUGUUUUCAGGAAGUAGUUUUUUAACAAAAGUCCUUCAUCAA	PS_tet_ (44)	59.0
		PO	

BTT, 5-benzylthio-1-H-tetrazole; Nt, nucleotide; PO, phosphodiester; PS, phosphorothioate; siRNA, small interfering RNA; tet, 1H-tetrazole; *T*_m_, melting temperature.

^*^Sequences of the Lin28 (si19-22) and p53 (si23-26) siRNAs: guide strand (upper); passenger strand (lower).

^†^Nucleotide linkages in the strands of the siRNAs.

^‡^Calculated *R*p content of the PS ORN, assuming it is composed of independently acting dinucleotides.
