# Supplementary material for: Stereochemical bias introduced during RNA synthesis modulates the activity of phosphorothioate siRNAs
Source: Nat Commun. 2015 Mar 6;6:6317. doi: 10.1038/ncomms7317 (PMC4366519; doi:10.1038/ncomms7317)
Supplement: Supplementary Information — Supplementary Figures 1-17, Supplementary Tables 1-5 and Supplementary Notes 1-2 [file ncomms7317-s1.pdf]

Supplementary Figures

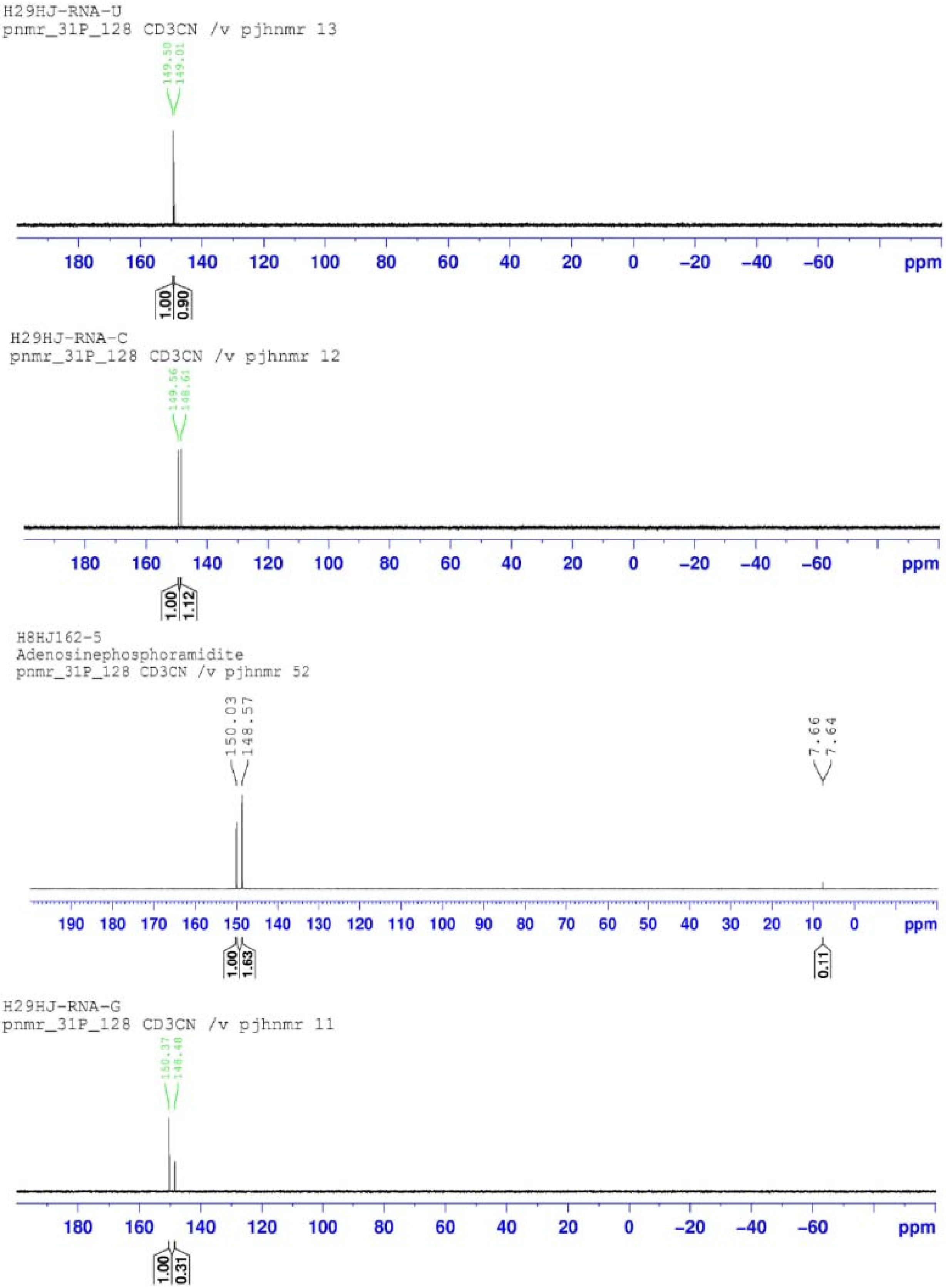

Supplementary Figure 1: ^{31}P-NMR spectra (d$_3$-ACN) of RNA-phosphoramidites showing 2 diastereomers (from top to bottom: uridine, cytidine, adenosine and guanosine.

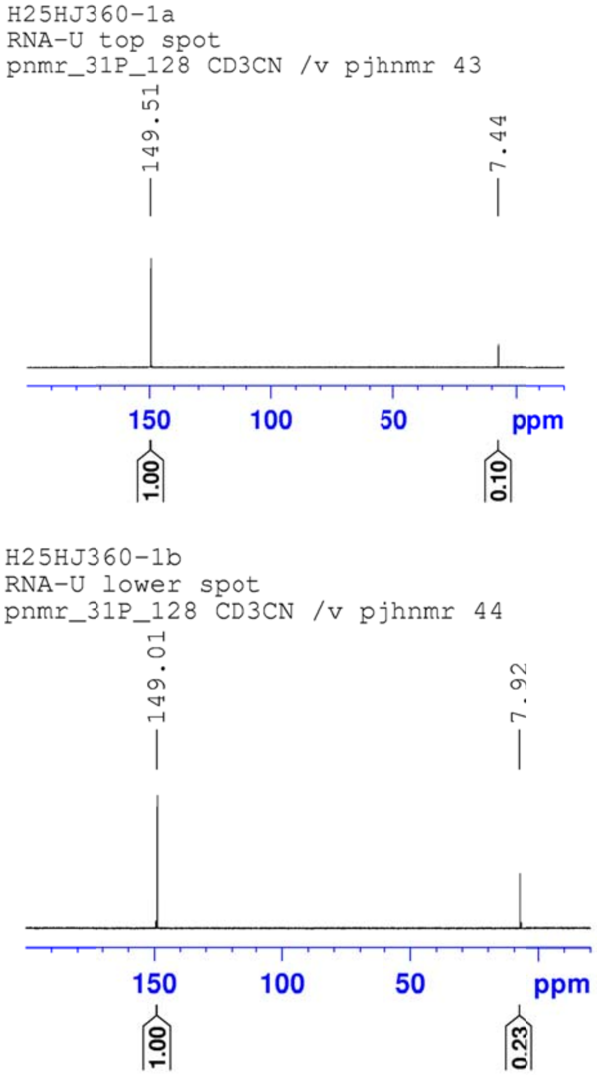

Supplementary Figure 2: ^{31}P-NMR spectra (d_3-ACN) of purified uridine-diastereomer-phosphoramidites: a) fast migrating (by chromatography) diastereomer, b) slow migrating diastereomer (by chromatography). Phosphoramidites diastereoisomers were separated by gravity silica gel column chromatography (Fluka; analytical grade: pore size 60 Å, 230-400 mesh particle size, high-purity grade (w/Ca,~0.1 %) using 1:1 n-hexane/ethylacetate + 2 % triethylamine. The peak at ~ 7 ppm represents a small amount of the H-phosphonate due to partial hydrolysis of the phosphoramidite.

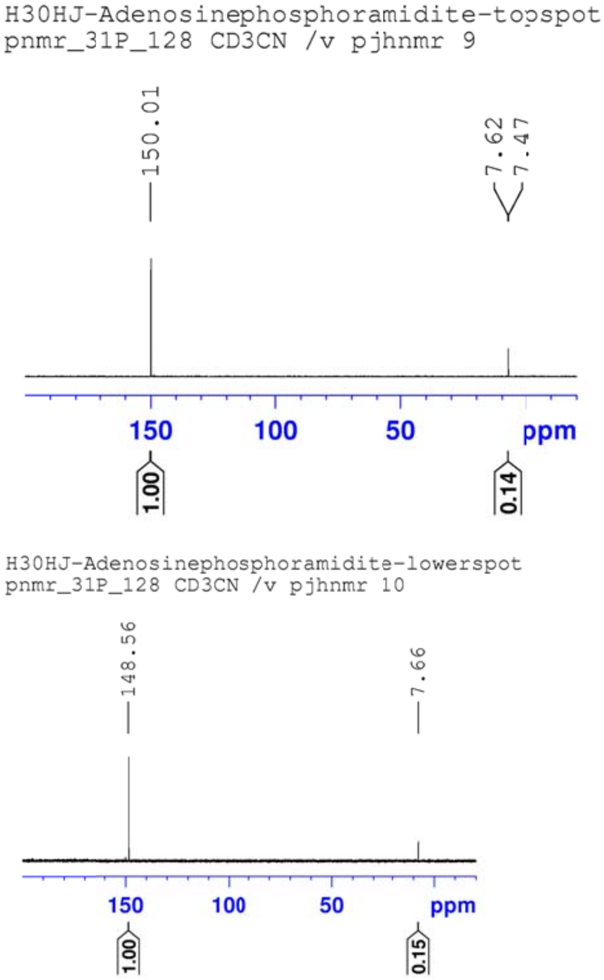

Supplementary Figure 3: ^{31}P-NMR spectra (d_3-ACN) of purified adenosine-diastereomer-phosphoramidites: a) fast migrating diastereomer (by chromatography), b) slow migrating diastereomer (by chromatography). Phosphoramidite diastereoisomers were separated by gravity silica gel column chromatography (Fluka; analytical grade: pore size 60 Å, 230-400 mesh particle size, high-purity grade (w/Ca,~0.1 %) using 1:1 n-hexane/ethylacetate + 2 % triethylamine. The peak at ~ 7 ppm represents the H-phosphonate due to partial hydrolysis of the phosphoramidite.

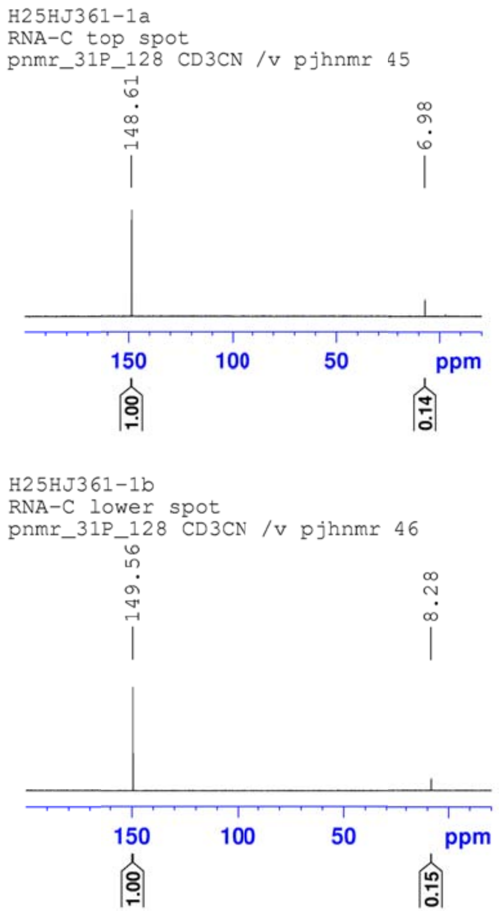

Supplementary Figure 4: ^{31}P-NMR spectra (d_3-ACN) of purified cytidine-diastereomer-phosphoramidites: a) fast migrating (by chromatography) diastereomer, b) slow migrating diastereomer (by chromatography). Phosphoramidite diastereoisomers were separated by gravity silica gel column chromatography (Fluka; analytical grade: pore size 60 Å, 230-400 mesh particle size, high-purity grade (w/Ca,~0.1 %) using 1:2 n-hexane/ethylacetate + 2 % triethylamine. The peak at ~ 7 ppm represents the H-phosphonate due to partial hydrolysis of the phosphoramidite.

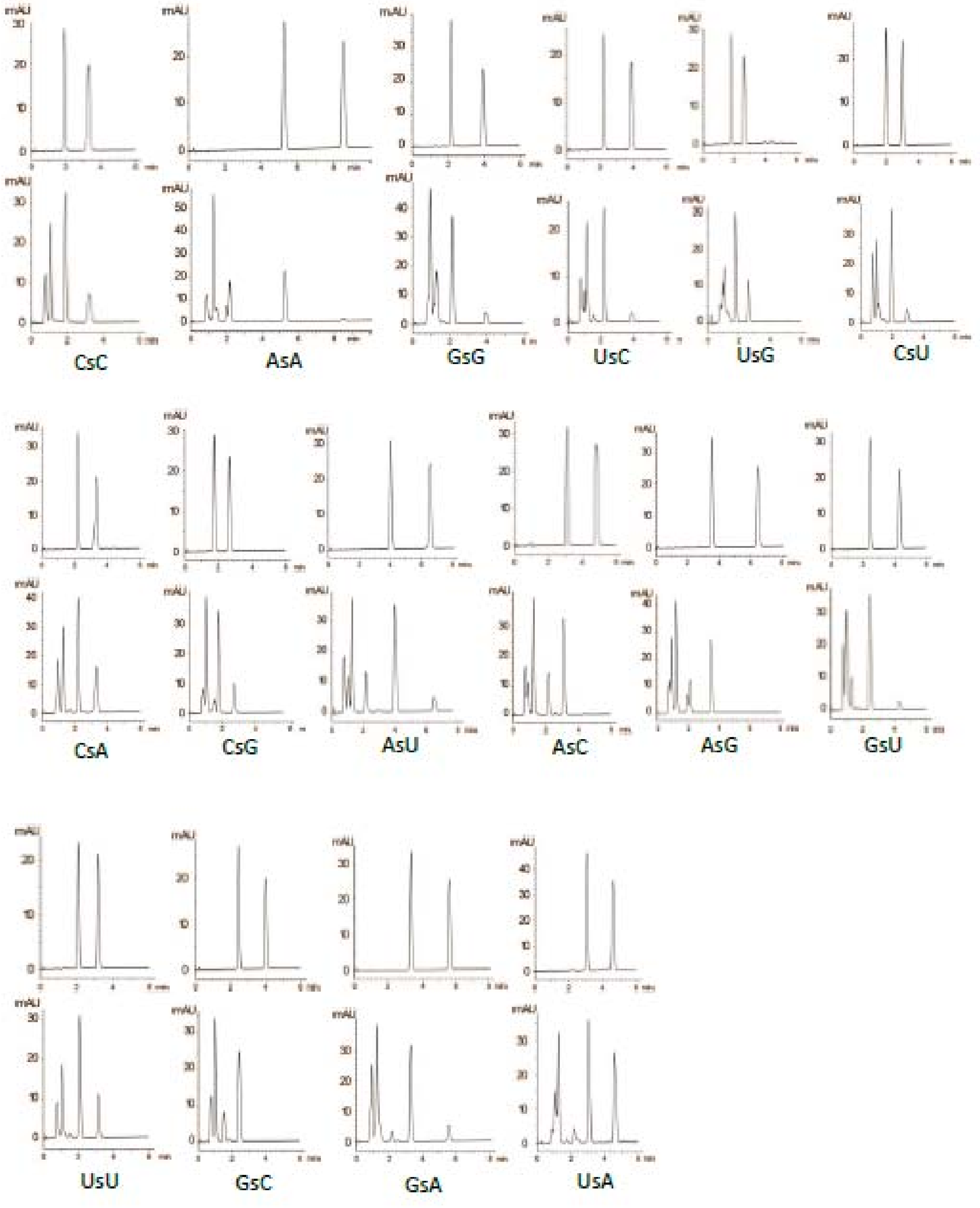

Supplementary Figure 5: Nuclease degradation assays. HPLC spectra of 16 phosphorothioate (PS)-diribonucleotides after incubation with nP1: upper chromatograms are reference spectra, lower chromatograms are obtained after incubation with nP1.

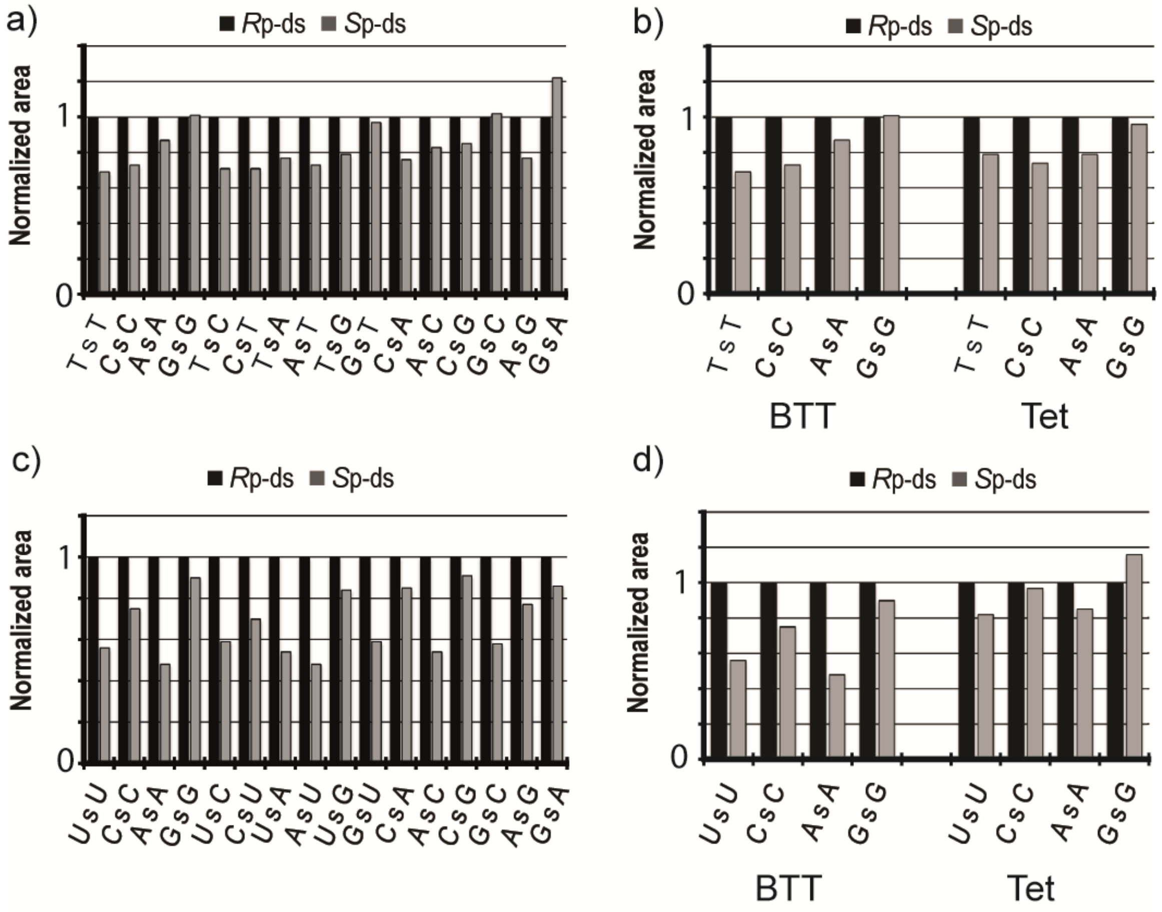

Supplementary Figure 6: Diastereoisomer ratios of PS-dinucleotides synthesized using BTT and tetrazole activators. DNA dinucleotides synthesized with BTT (a) and BTT and Tet (b). 2'-*O*-Me dinucleotides synthesized with BTT (c) and BTT and Tet (d) , as described in Figure 3a. (Syntheses of the UsU, CsC, GsG and AsA 2'-OMe dinucleotides with BTT (shown in d)) were performed at least 8 times with almost no variations found in the ratios).

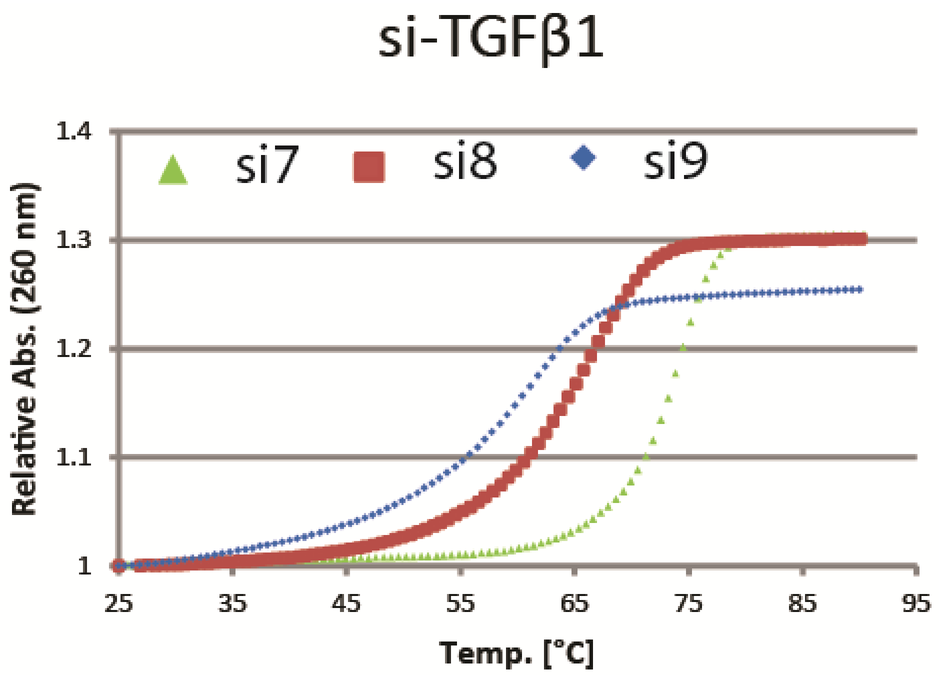

Supplementary Figure 7: Melting curves of PO- and PS- (BTT, Tet) siTGFβ-1: si7, si8 and si9. Oligonucleotide concentrations were determined by measuring the absorbance on a *Cary* 300 Bio UV spectrometer (Varian) at 260 nm and dividing the obtained values by the calculated extinctions coefficients (nearest neighbor method according to Owczarzy (Biophysical Chemistry, 2008, Vol. 133, No. 1-3, pp 66-70)). Melting curves for the duplexes in Tables 1 and 2 were measured on a *Cary* 300 Bio UV spectrometer (Varian) equipped with a thermoelectrical controller using a concentration of 2 µM in Na_2HPO_4 (2.5 mM), $NaH_2PO_4{\cdot}H_2O$ (5 mM), NaCl (100 mM) and $Na_2EDTA{\cdot}2H_2O$ (0.1 mM) buffer, pH 6.4. The change in absorbance at 260 nm as a function of time was recorded for the duplexes in Tables 1 and Table 2 while the temperature was decreased/increased at a cooling/heating rate of 0.5°C min^{-1} under standard conditions. T_m was determined by taking the local maximum of the first derivatives of the absorbance vs. temperature curve. All melting curves measurements were performed in triplicates (Supplementary Figures 7-10).

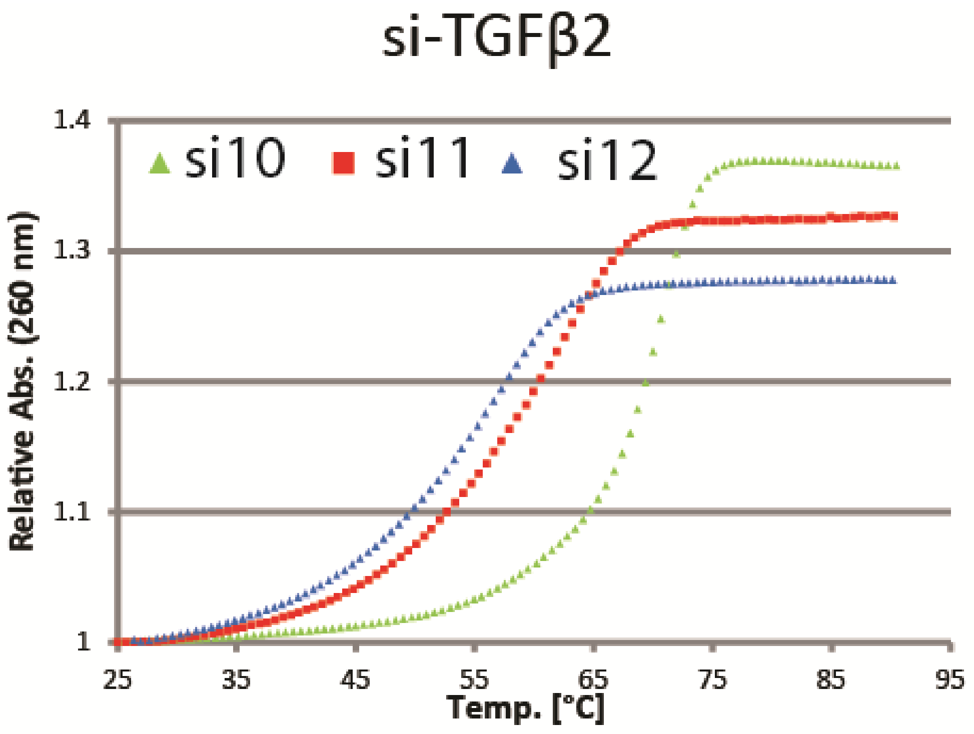

Supplementary Figure 8: Melting curves of PO- and PS- (BTT, Tet) siTGFβ-2: si10, si11 and si12. Method is described in the legend of Supplementary Figure 7.

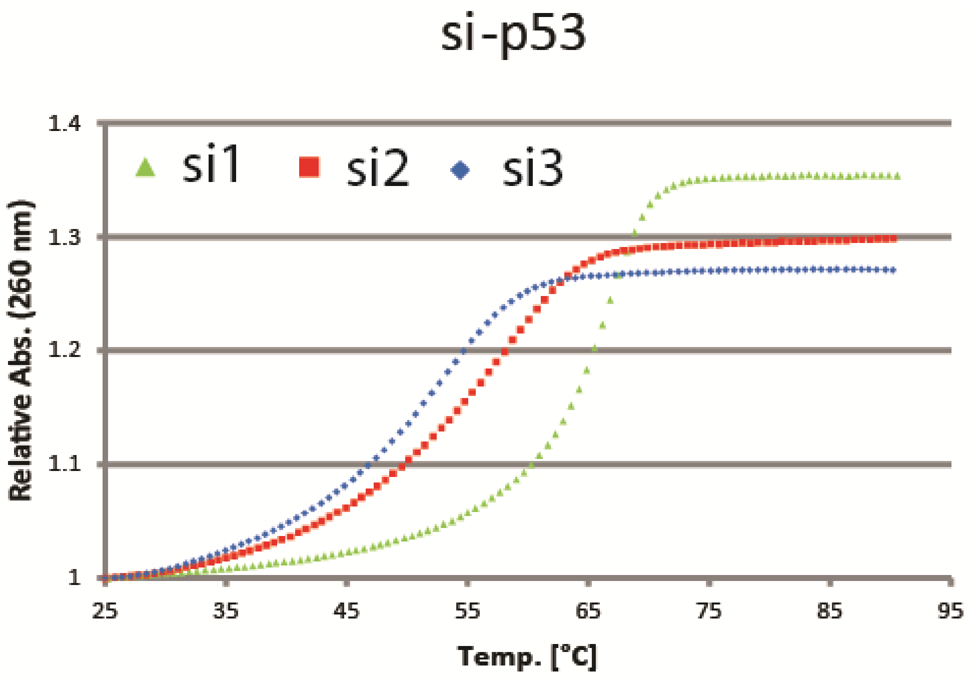

Supplementary Figure 9: Melting curves of PO- and PS- (BTT, Tet) si-p53: si1, si2 and si3. Method is described in the legend of Supplementary Figure 7.

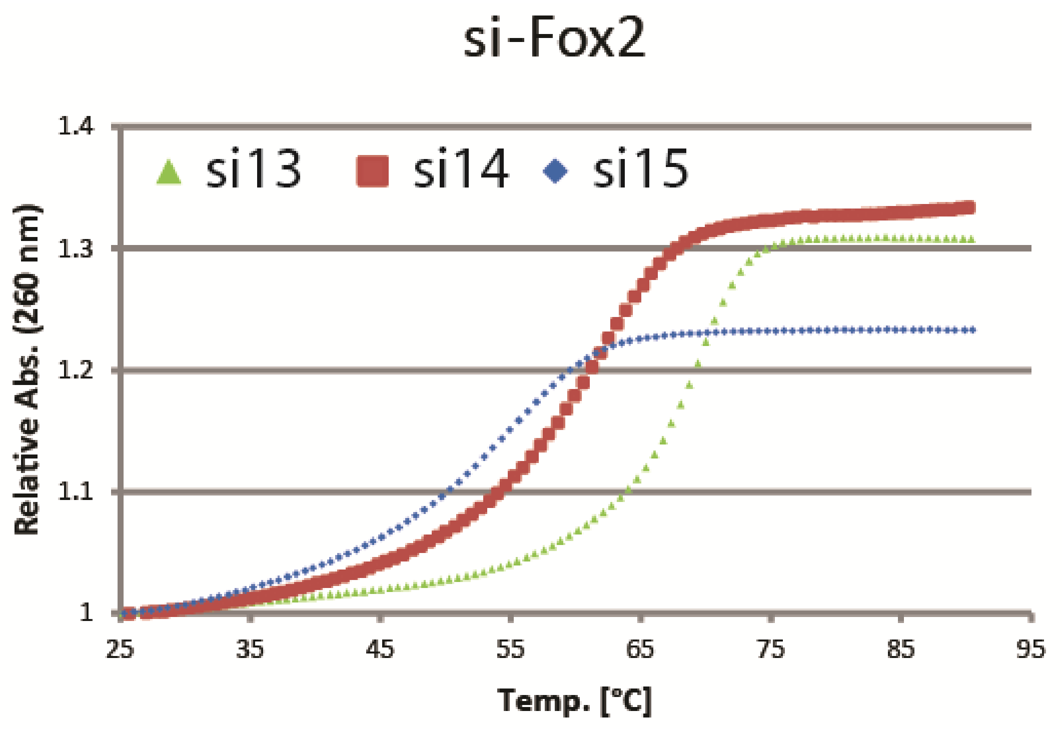

Supplementary Figure 10: Melting curves of PO- and PS- (BTT, Tet) si-Fox2: si13, si14 and si15. Method is described in the legend of Supplementary Figure 7.

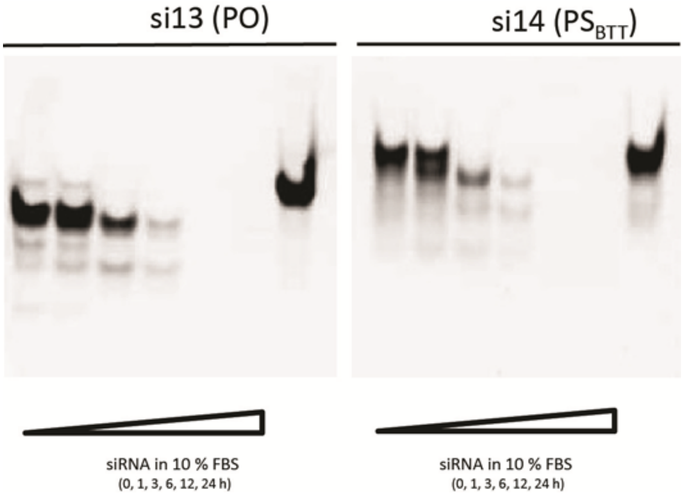

Supplementary Figure 11: Stability of siFox2 (si13 and si14) in 10 % FBS (PO: both strands of siRNA are phosphodiesters; PS_{BTT}: both strands of siRNA are phosphorothioates synthesized with BTT): lanes 1-6 from left to right are: 0 h (sample assayed directly after FBS addition), 1 h, 3 h, 6 h, 12 h and 24 h. Lane 7 contains siRNA with no added FBS.

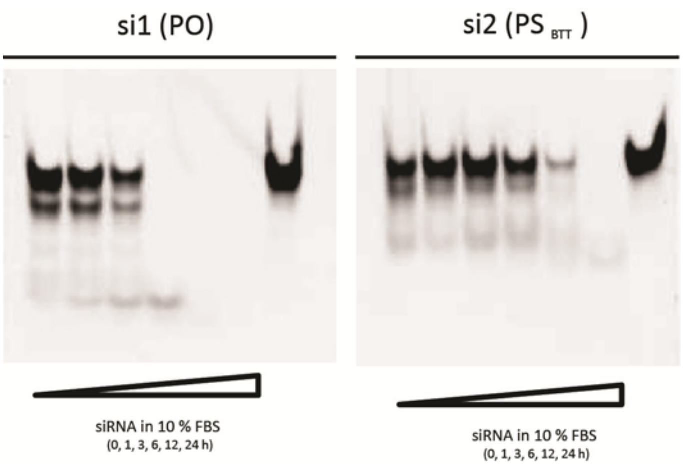

Supplementary Figure 12: Stability of sip53 (si1 and si2) in 10 % FBS (PO: both strands of siRNA are phosphodiesters; PS_{BTT}: both strands of siRNA are phosphorothioates synthesized with BTT): lanes 1-6 from left to right are: 0 h (sample assayed directly after FBS addition), 1 h, 3 h, 6 h, 12 h and 24 h. Lane 7 contains siRNA with no FBS.

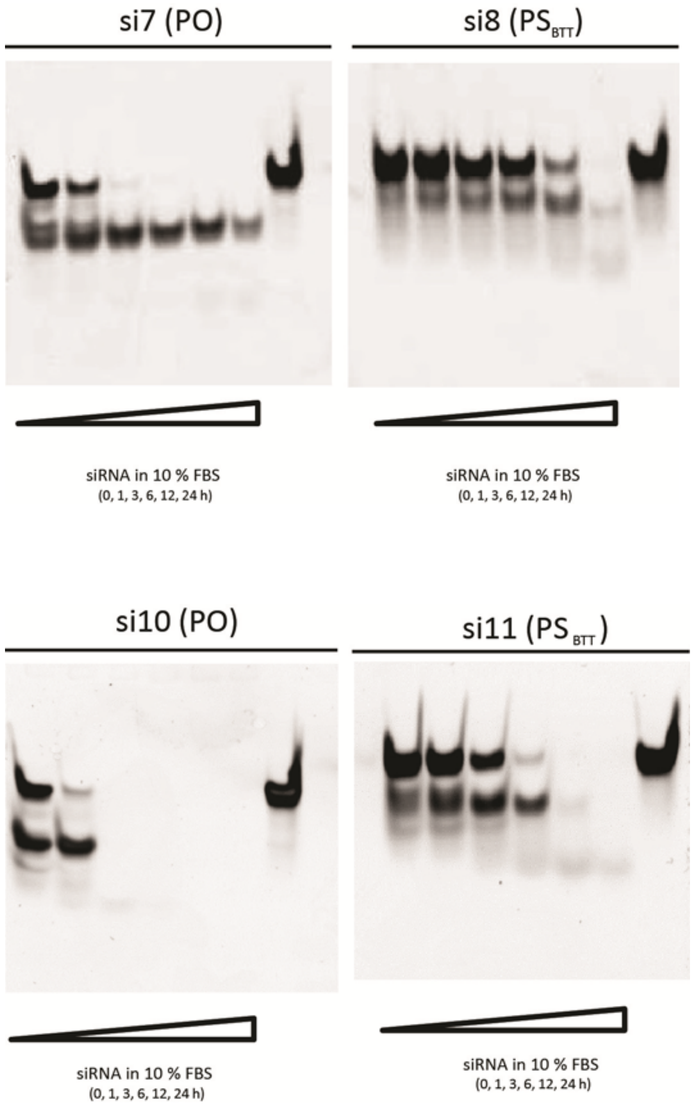

Supplementary Figure 13: Stability of siTGFβ-1 (si7 and si8) and siTGFβ-2 (si10 and si11) in 10 % FBS (PO: both strands of siRNA are phosphodiesters; PS_{BTT}: both strands of siRNA are phosphorothioates synthesized with BTT): lanes 1-6 from left to right are: 0 h (sample assayed directly after FBS addition), 1h, 3 h, 6 h, 12 h and 24 h. Lane 7 contains siRNA with no FBS.

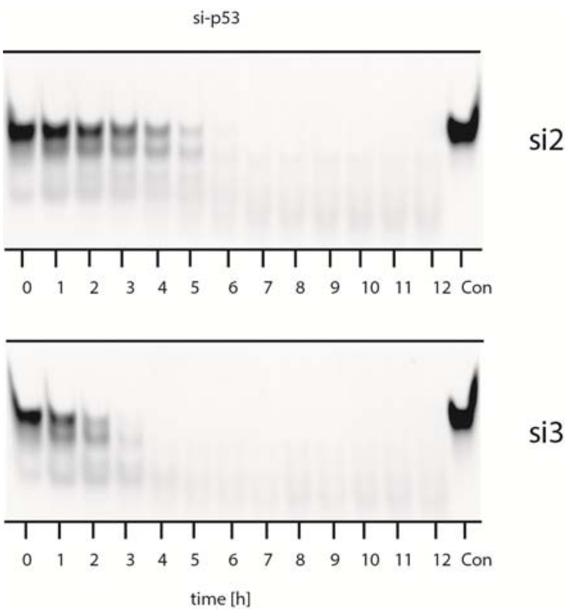

Supplementary Figure 14: Stability after incubation in 10 % FBS of siP53 siRNAs si2 and si3 (top gel: si2 , PS_{BTT} synthesized; bottom gel: si3 PS_{Tet} synthesized): lanes 1-13 from left to right are: 0 h (sample assayed directly after FBS addition), 1 h, 2 h, 3 h, 4 h, 5 h, 6 h, 7 h, 8 h, 9 h, 10 h, 11 h and 12 h. Lane 13 contains siRNA with no FBS.

a)

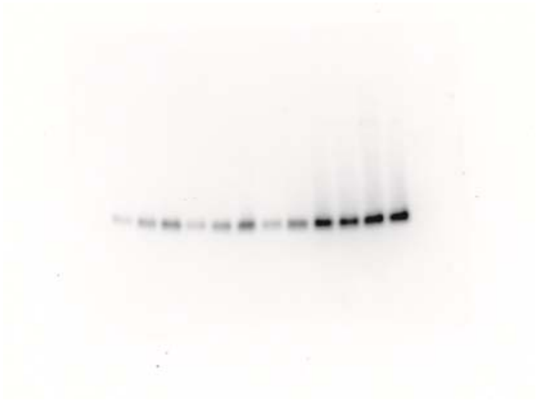

b)

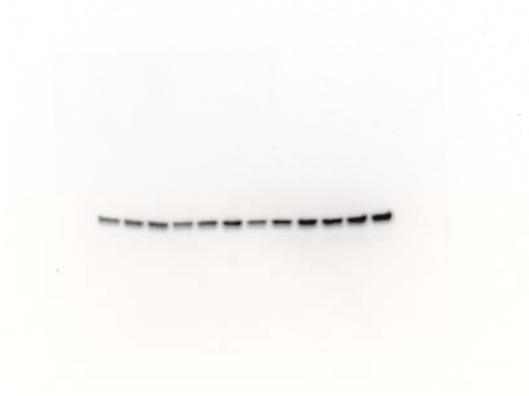

c)

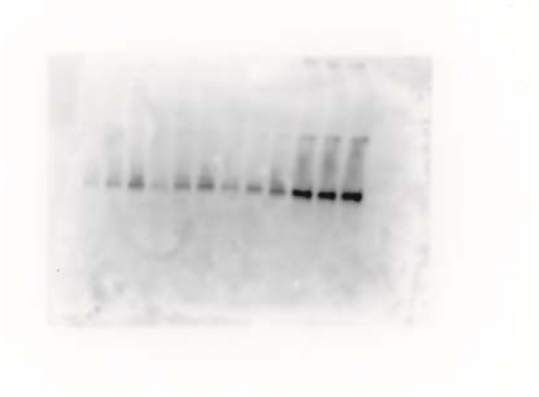

d)

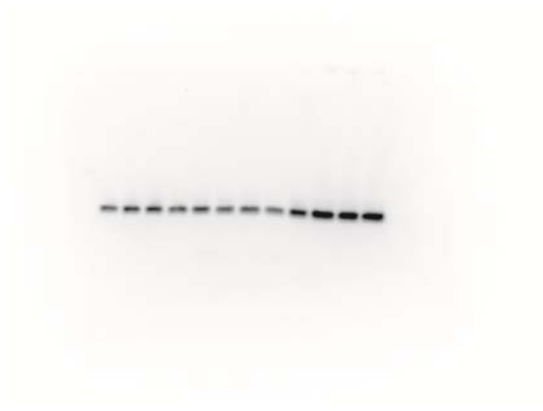

Supplementary Figure 15: Uncropped western blots shown in the manuscript. a) and b) Lin28 and beta-actin protein respectively, after treatment with PO- and PS-siRNAs in Huh7 cells (24, 6, 2.5 nM) shown in Fig 4c. c) and d) p53 and beta actin protein, respectively, in HeLa cells after treatments with 6, 2.5, and 0.6 nM siRNAs.

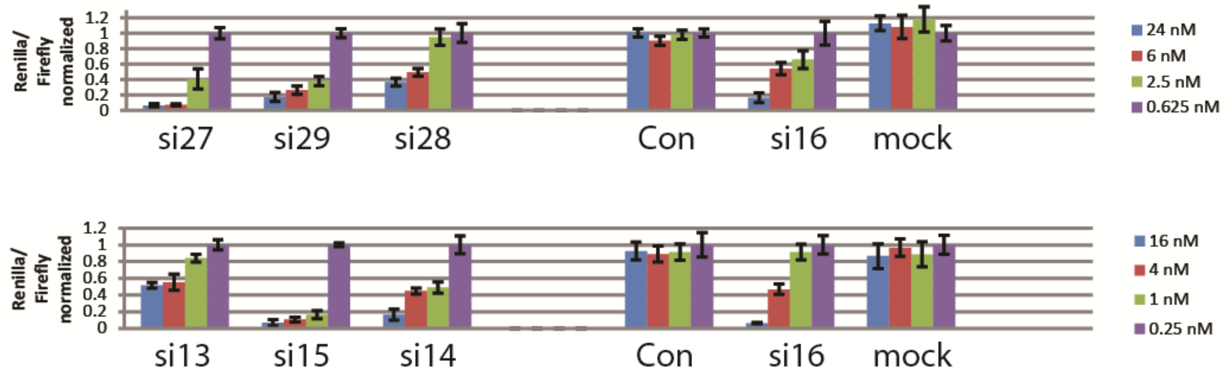

Supplementary Figure 16: Sequence-specific inhibition of *Renilla*/firefly luciferase reporters in HeLa Cells. Upper panel: siRNAs targeting Fox1 reporter (sequences in Supplementary Table 5); Lower panel: siRNAs targeting Fox2 reporter (sequences in Supplementary Table 5).

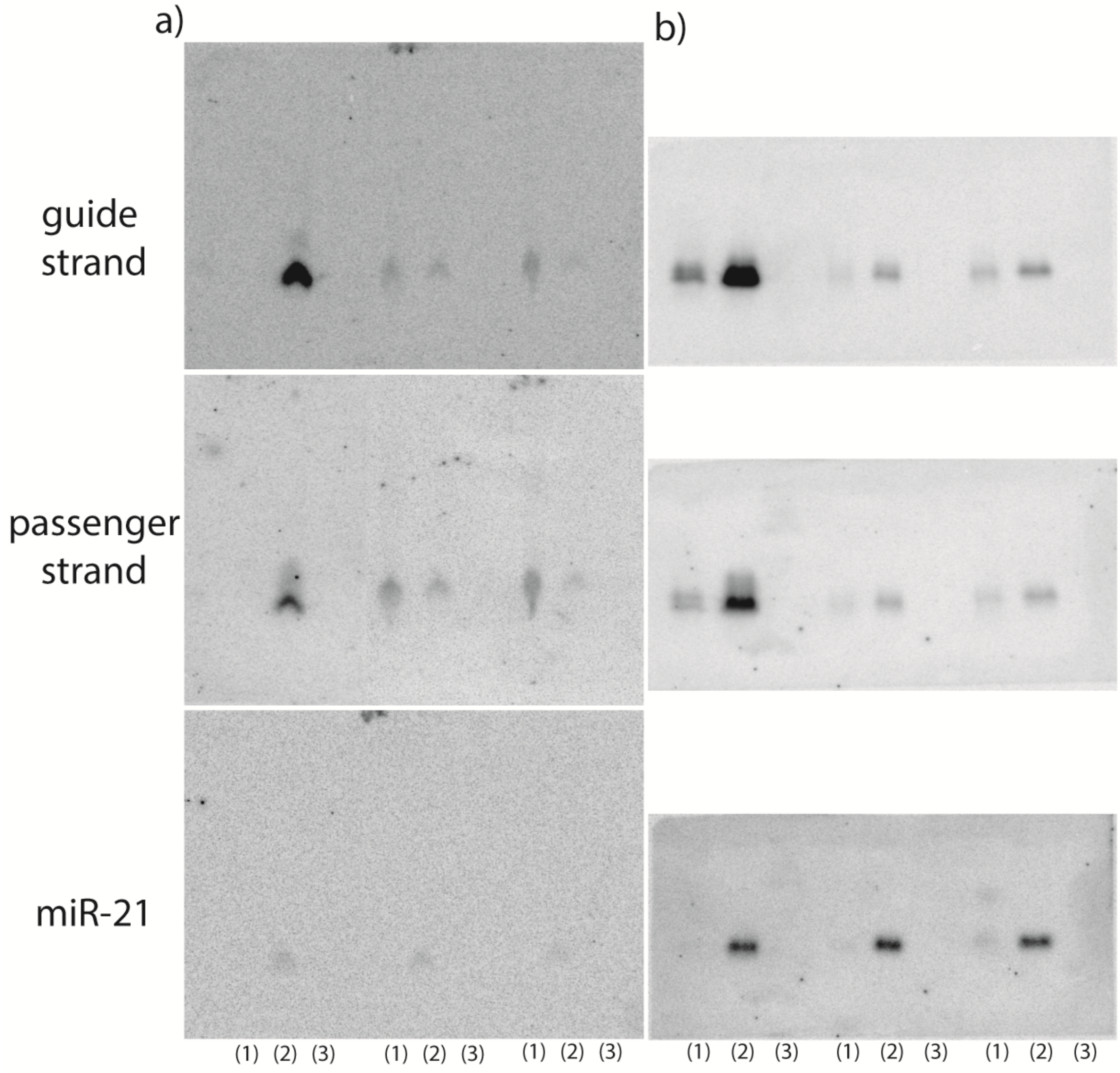

Supplementary Figure 17. Northern blots of RISC isolated from transfected HeLa cells showing guide and passenger strand composition. Composition of RISC (Ago2) from two independent experiments (a) and b)) from HeLa cells transfected (30 nM) with PO-, PS_{BTT}- and PS_{tet}-siRNAs (si1, si2, si3, resp.) and worked up under identical conditions after 24 h. Replicate b) is shown in the manuscript Figure 6. Northern blot detection using ^{32}P-labeled respective counter-strand as detection probe of guide (upper blot) and passenger (center blot) strands of si1 (left panel), si2 (center panel) and si3 (right panel). RNA samples loaded were Input RNA (5 % of total, Rep a), Ago2 and control immunoprecipitation (Ø-IP) (each 47.5%). Endogenously-expressed miR-21 detection (lower blot) served as a loading control. (Lane (1): Input, 5%; Lane (2): Ago2-IP, 47.5%; Lane (3): Control IP, 47.5%).

Supplementary Tables

Supplementary Table 1: Chemical shifts [ppm] and ratios of the diastereomer-phosphoramidites

Phosphoramidite	Chemical shift [ppm]	Integration Peak1	Integration Peak2	Ratio
Uridine	149.50/149.01	1	0.9	1.11
Cytidine	149.56/148.61	1	1.12	0.89
Adenosine	150.03/148.57	1	1.63	0.61
Guanosine	150.37/148.48	1	0.3	0.333

Supplementary Table 2: Properties of diribonucleotides synthesized with BTT as activator analyzed by RP-HPLC (sequence, Rp-Sp-ratio (ds = diasteromer), row value of HPLC area under the peak, retention time (r.t.) of the different diastereomers, observed mass and HPLC running buffer conditions). The 16 phosphorothioate (PS) diastereomeric diribonucleotides were synthesized DMT-off, separated by RP-HPLC and analyzed for their masses. For RP-HPLC purification/separation of dinucleotides and octanucleotides diastereomers: Buffer A: 0.1 M aqueous ammonium acetate, pH 7.0; buffer B: 20 % buffer A and 80 % ACN; flow-rate: 4 mL/min.

sequence	Rp-ds normalized	Sp-ds normalized	diastereomer 1 [mAU*s]	diastereomer 2 [mAU*s]	r.t. diastereomer 1 [min]	r.t. diastereomer 2 [min]	mass calc. [g/mol]	mass obs. [g/mol]	buffer B [%]
U s U	1	0.38	6015.10	2314.28	2.2	3.4	566.4	565.1	1-5
C s C	1	0.7	2413.95	1681.78	1.8	2.7	564.5	563.1	0.5-3
A s A	1	0.84	5988.79	5049.35	5.4	9.0	612.5	611.1	2-7
G s G	1	1.06	7008.88	7405.64	2.2	4.3	644.5	643.1	2-7
U s C	1	0.47	3150.88	1487.47	2.3	4.1	565.5	564.1	1-5
C s U	1	0.53	3768.23	1980.95	2.1	3.2	565.5	564.1	1-5
U s A	1	0.68	4339.44	2954.24	3.2	4.8	589.5	588.1	2-7
A s U	1	0.65	6338.46	4140.87	4.3	6.8	589.5	588.2	2-7
U s G	1	0.6	6016.89	3605.09	1.9	2.8	605.5	604.2	2-7
G s U	1	0.71	5878.78	4141.89	2.6	4.5	605.5	604.1	2-7
C s A	1	0.7	4075.01	2843.77	2.2	3.6	588.5	587.1	2-7
A s C	1	0.61	4378.52	2651.69	3.3	5.1	588.5	587.1	2-7
C s G	1	0.7	6601.12	4644.07	1.9	2.8	604.5	603.1	1-4
G s C	1	0.44	1621.11	717.25	2.5	4.2	604.5	603.1	1-5
A s G	1	0.77	8239.52	6321.33	3.7	5.7	628.5	627.1	2-7
G s A	1	0.8	5465.83	4363.82	3.5	5.5	628.5	627.2	2-7

Supplementary Table 3: Properties of diribonucleotides synthesized with tetrazole as activator and anylyzed by RP-HPLC (sequence, Rp-Sp-ratio (ds = diasteromer), row value of HPLC area under the peak, retention time (r.t.) of the different diastereomers, observed mass and HPLC running buffer conditions)

sequence	Rp-ds normalized	Sp-ds normalized	diastereomer 1 [mAU*s]	diastereomer 2 [mAU*s]	r.t. diastereomer 1 [min]	r.t. diastereomer 2 [min]	mass calc. [g/mol]	mass obs. [g/mol]	buffer B [%]
U s U	1	1.13	10641.0	12025.1	2.8	4.6	566.4	565.1	1-5
C s C	1	1.39	6786.8	9431.9	2.0	3.3	564.5	563.1	0.5-3
A s A	1	1.2	32820.5	36316.3	4.9	8.1	612.5	611.1	2-7
G s G	1	1.83	16159.2	29520.9	2.1	4.1	644.5	643.1	2-7
U s C	1	1.19	13485.6	16107.7	2.2	4.0	565.5	564.1	1-5
C s U	1	1.16	15800.7	18371.2	1.8	2.4	565.5	564.1	1-5
U s A	1	1.11	17664.8	19612.7	2.8	4.1	589.5	588.1	2-7
A s U	1	1.04	15642.1	16233.7	4.1	6.0	589.5	588.2	2-7
U s G	1	1.22	16796.5	20440.4	1.6	2.2	605.5	604.2	2-7
G s U	1	1.78	15090.2	26837.4	2.5	4.3	605.5	604.1	2-7
C s A	1	0.91	21349.0	19481.4	1.9	2.9	588.5	587.1	2-7
A s C	1	1.19	14878.1	17691.5	3.2	4.9	588.5	587.1	2-7
C s G	1	1.11	14567.2	16193.4	1.9	2.6	604.5	603.1	1-4
G s C	1	1.6	14219.8	22777.3	2.3	4.2	604.5	603.1	1-5
A s G	1	1.3	25708.5	33441.8	3.7	6.2	628.5	627.1	2-7
G s A	1	1.98	17785.0	35164.7	3.4	5.4	628.5	627.2	2-7

Supplementary Table 4: Properties of octaribonucleotides anylzed by RP-HPLC (sequence, normalized ratio of diastereomers (Rp-ds and Sp-ds), raw value of HPLC area under the peak of the diastereomers (diastereomer 1 = fast migrating on HPLC, diastereomer 2 = slow migrating on HPLC), retention time (r.t.) of the diastereomers, observed mass and calculated mass

SEQUENCE	Rp-ds normalized	Sp-ds normalized	Diastereomer 1 [mAU*s]	Diastereomer 2 [mAU*s]	r.t. diastereomer 1 [min]	r.t. Diastereomer 2 [min]	obs. Mass [g/mol]	calc. Mass [g/mol]
UUUUsUUUU	1	0.38	11906.30	4569.59	3.3	5.0	2403.5	2403.28
UCAUsUCGU	1	0.38	9823.18	3719.94	3.9	5.2	2462.83	2463.6
UCAUUCGsU	1	0.6	14254.06	8540.31	6.7	8.2	2463.23	2463.6
UsCAUUCGU	1	0.53	16334.90	8654.19	3.0	5.7	2463.23	2463.6
UsUAUUCGU	1	0.22	11983.90	2641.61	4.7	7.9	2464.03	2464.6
UCAUUCUsU	1	0.39	1403.93	547.47	5.2	6.2	2424.43	2424.5
AAAAsAAAA	1	0.79	20890.67	16527.75	3.2	4.1	2587.8	2587.25
CCCCsCCCC	1	0.55	7494.99	4145.71	2.1	4.9	2395.5	2394.72

Supplementary Table 5: Composition and properties for PO- and PS-ORNs: activator used in ORN synthesis, sequence of guide and passenger strands, mass calculated and mass observed

name of siRNA	si-target	backbone modification	activator	ORN sequence 5' to 3'	mass calc.	mass obs.
si1	P53	all PO		AACUACUUCCUGAAAACAAUU	6609,1	6607,9
		all PO		UUGUUUUCAGGAAGUAGUUUU	6654,0	6653,0
si2		all PS	Tet	AACUACUUCCUGAAAACAAUU	6930,4	6930,9
		all PS	Tet	UUGUUUUCAGGAAGUAGUUUU	6975,3	6976,1
si3		all PS	BTT	AACUACUUCCUGAAAACAAUU	6930,4	6929,5
		all PS	BTT	UUGUUUUCAGGAAGUAGUUUU	6975,3	6974,1
si4	Lin28	all PO		AAAUCCUUCCAUGAAUAGUTT	6599,1	6598,2
		all PO		ACUAUUCAUGGAAGGAUUUTT	6656,1	6655,3
si5		all PS	Tet	AAAUCCUUCCAUGAAUAGUTT	6918,2	6919,2
		all PS	Tet	ACUAUUCAUGGAAGGAUUUTT	6976,1	6976,4
si6		all PS	BTT	AAAUCCUUCCAUGAAUAGUTT	6918,2	6919,3
		all PS	BTT	ACUAUUCAUGGAAGGAUUUTT	6976,1	6976,5
si7	TGFβ1	all PO		CCAACUAUUGCUUCAGCUCUU	6530,9	6530,3
		all PO		GAGCUGAAGCAAUAGUUGGUU	6777,1	6776,4
si8		all PS	Tet	CCAACUAUUGCUUCAGCUCUU	6852,2	6851,1
		all PS	Tet	GAGCUGAAGCAAUAGUUGGUU	7098,5	7097,2
si9		all PS	BTT	CCAACUAUUGCUUCAGCUCUU	6852,2	6851,2
		all PS	BTT	GAGCUGAAGCAAUAGUUGGUU	7098,5	7097,3
si10	TGFβ2	all PO		GGAUUGAGCUAUAUCAGAUUUU	7005,2	7004,7
		all PO		AAUCUGAUAUAGCUCAAUCCUU	6908,2	6905,9
si11		all PS	Tet	GGAUUGAGCUAUAUCAGAUUUU	7342,6	7341,8
		all PS	Tet	AAUCUGAUAUAGCUCAAUCCUU	7245,6	7244,3
si12		all PS	BTT	GGAUUGAGCUAUAUCAGAUUUU	7342,6	7341,6
		all PS	BTT	AAUCUGAUAUAGCUCAAUCCUU	7245,6	7244,3
si13	Fox2	all PO		CCUGGCUAUUGCAAUAUUUUU	6572,9	6571,9
		all PO		AAAUAUUGCAAUAGCCAGGUU	6705,1	6704,3
si14		all PS	Tet	CCUGGCUAUUGCAAUAUUUUU	6894,2	6895,4
		all PS	Tet	AAAUAUUGCAAUAGCCAGGUU	7026,4	7024,4
si15		all PS	BTT	CCUGGCUAUUGCAAUAUUUUU	6894,2	6893,1
		all PS	BTT	AAAUAUUGCAAUAGCCAGGUU	7026,4	7025,6
si16	Ren	all PO		GAGCGAAGAGGGCGAGAAAUU	6901,3	6900,0
		all PO		AAUUUCUCGCCCUCUUCGCUC	6482,9	6481,9
si17		all PS	Tet	GAGCGAAGAGGGCGAGAAAUU	7222,6	7221,5
		all PS	Tet	AAUUUCUCGCCCUCUUCGCUC	6804,2	6803,2
si18		all PS	BTT	GAGCGAAGAGGGCGAGAAAUU	7222,6	7221,2
		all PS	BTT	AAUUUCUCGCCCUCUUCGCUC	6804,2	6803,4
si27	Fox1	all PO		GAUUUGGUUUCGUAACUUUUU	6590,9	6591,1
		all PO		AAAGUUACGAAACCAAAUCUU	6672,1	6672,0
si28		all PS	Tet	GAUUUGGUUUCGUAACUUUUU	6912,2	6913,1
		all PS	Tet	AAAGUUACGAAACCAAAUCUU	6993,4	6993,1
si29		all PS	BTT	GAUUUGGUUUCGUAACUUUUU	6912,2	6911,2
		all PS	BTT	AAAGUUACGAAACCAAAUCUU	6993,4	6992,3
pos	Renilla	all PO		GAGCGAAGAGGGCGAGAAAUU	-	-
		all PO		UUUCUCGCCCUCUUCGCUCUU	-	-

Supplementary Notes

Supplementary Note 1.

Reproducibility of the Rp/Sp ratios in dinucleotide couplings. The dinucleotide experiments shown in Figures 2c and 2d (UsU, CsC, AsA) were carried out several times in replicate experiments using various sulfurization reagents over many months using different batches of phosphoramidites and solid supports. The replicate experiments are summarized below.

- Fig 2c “Mixture” was performed independently 5 times with DDTT and produced ratios of 0.38, 0.52, 0.52, 0.52 and 0.52.
- Fig 2d right panel: “Mixture” was done twice with DDTT and gave 0.85 and 0.85.
- Fig 2d right panel: “Fast” was done twice with DDTT and gave 0.70 and 0.92.

We found that the isomeric product ratios of Fig 1 were not influenced by different sulfurization reagents, as expected from previous literature reports (data not shown).

- Fig 2c “Mixture” was performed with PADS, ADTT and Beaucage sulfurization reagents and gave 0.44, 0.43 and 0.41, respectively
- Fig 2d left panel: “Mixture” was performed with PADS and ADTT sulfurization reagents the values were 0.79 and 0.72, respectively
- Fig 2d right panel: “Mixture” was performed with PADS and ADTT sulfurization reagents the values were 0.78 and 0.87, respectively

Supplementary Note 2.

Reporter plasmid sequences. The inserted sequences in the psiCHECK2 vector were sequenced and are reported below.

- p53 (for si1, si2 and si3)
  CCTCCACTTCAGCCAGGACTCGAGTCCTAGCGTCGAGCCCCCTCTGAGTCAGGAAACATTTTCAGACC
  TATGGAAACTACTTCCTGAAAACAACGTTCTGTCCCCCTTGCCGTCCCAAGCAATGGATGATTTGATG
  CTGTCCC GGCCGC TGAGTCTTCGGACCTCGC
- Fox-2 (for si13, si14 and si15)
  CCTCCACTTCAGCCAGGA CTCGAG TGAAGTGACGTGAGACCCCTGCAAATGGGACAGCCCCCC
  AGTTCATGAGG CCTGGCTATTGCAATATTT ACTAGTAGAGGAACTCTATAGCAAGATGAAGAGGA
  AAAACAAACAAACAA GGCCGC TGAGTCTTCGGACCTCGC
- Fox-1 (for siFox1, BTT and Tet)
  CCTCCACTTCAGCCAGGA CTCGAG TGGTAAAATCTTAGATGTTGAAATTATTTTTAATGAGCGA
  GGCTCAAAGG GATTTGGTTTCGTAACTTT CGAAAATAGTGCCGATGCGGACAGGGCGAGGGAGA
  AATTACACGGCACCG GGCCGC TGAGTCTTCGGACCTCGC
- TGFbeta1 (for si7, si8 and si9)
  CCTCCACTTCAGCCAGGACTCGAGGGAGAGGGCCCAGCATCTGCAAAGCTCCCGGCACCGCCGAGCC
  CTGGACACCAACTATTGCTTCAGCTCCACGGAGAAGAACTGCTGCGTGCGGCAGCTGTACATTGACTT
  CCGCAAGG GGCCGC TGAGTCTTCGGACCTCGC
- LIN28B (for si4, si5 and si6)
  CCTCCACTTCAGCCAGGACTCGAGGGGGCGCTATTCATGGAAGGATTTAGCAGCAGTTGGATCTTTTG
  AAGGGAGAAGACACTGCAGTGACCACTTATTCTGTATTGCCATGGTCTTTCCACTTTCATCTGGGGTG
  GGGTGGGGTGGGGTGGGGGAGGGGGGGGTGGGGTGGCGGCCGCTGAGTCTTCGGACCTCGC
- Renilla (for si16, si17 and si18)
  empty psi-Check-2 vector (psiCHECK™-2, Promega)
